# NMR Reveals Functionally Relevant Thermally Induced Structural Changes within the Native Ensemble of G-CSF

**DOI:** 10.1021/acs.molpharmaceut.2c00398

**Published:** 2022-08-10

**Authors:** Mark-Adam W. Kellerman, Teresa Almeida, Timothy R. Rudd, Paul Matejtschuk, Paul A. Dalby

**Affiliations:** † Department of Biochemical Engineering, University College London, Gower Street, London WC1E 6BT, United Kingdom; ‡ Medicines & Healthcare Products Regulatory Agency, National Institute for Biological Standards and Control (NIBSC), Blanche Lane, South Mimms, Potters Bar, Hertfordshire EN6 3QG, United Kingdom; § Department of Biochemistry and Systems Biology, Institute of Systems, Molecular and Integrative Biology, University of Liverpool, Liverpool L69 7BE, United Kingdom

**Keywords:** denaturation, remodeling, structure−function, “switch”, aggregation

## Abstract

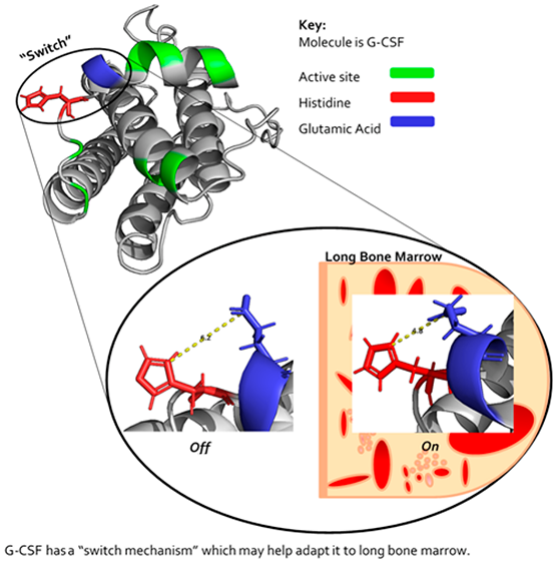

Structure–function relationships in proteins refer to a trade-off between stability and bioactivity, molded by evolution of the molecule. Identifying which protein amino acid residues jeopardize global or local stability for the benefit of bioactivity would reveal residues pivotal to this structure–function trade-off. Here, we use ^15^N–^1^H heteronuclear single quantum coherence (HSQC) nuclear magnetic resonance (NMR) spectroscopy to probe the microenvironment and dynamics of residues in granulocyte colony-stimulating factor (G-CSF) through thermal perturbation. From this analysis, we identified four residues (G4, A6, T133, and Q134) that we classed as significant to global stability, given that they all experienced large environmental and dynamic changes and were closely correlated to each other in their NMR characteristics. Additionally, we observe that roughly four structural clusters are subject to localized conformational changes or partial unfolding prior to global unfolding at higher temperature. Combining NMR observables with structure relaxation methods reveals that these structural clusters concentrate around loop AB (binding site III inclusive). This loop has been previously implicated in conformational changes that result in an aggregation prone state of G-CSF. Residues H43, V48, and S63 appear to be pivotal to an opening motion of loop AB, a change that is possibly also important for function. Hence, we present here an approach to profiling residues in order to highlight their potential roles in the two vital characteristics of proteins: stability and bioactivity.

## 1 Introduction

**Figure 1:**
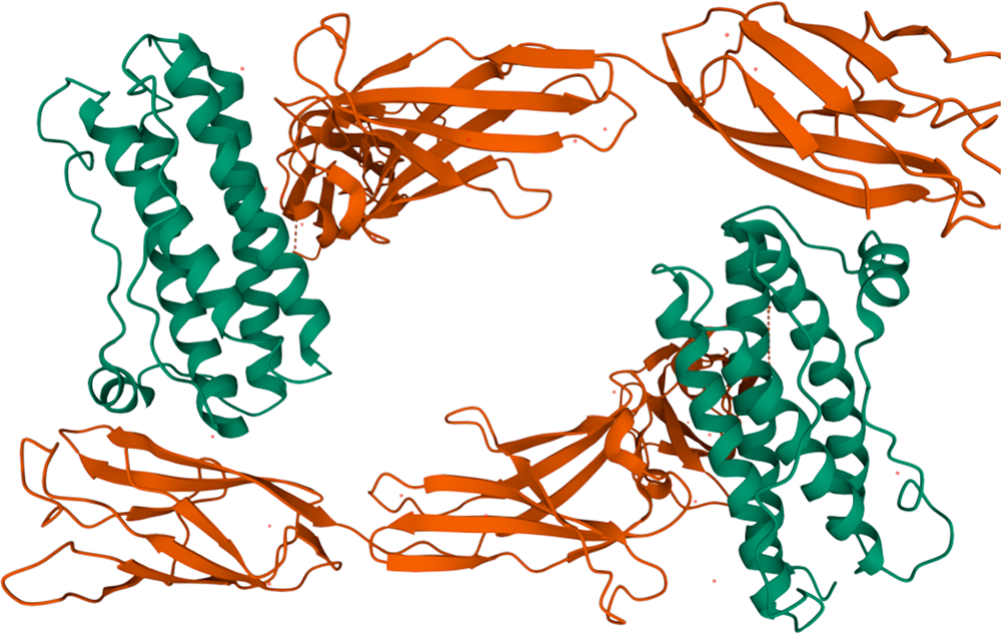
G-CSF in complex with its receptor crystal structure of two G-CSF molecules (green) in complex two G-CSF receptors (orange).^3^

The principal immune-regulatory cytokine in neutrophil development and function is granulocyte colony-stimulating factor (G-CSF).^1^ The multiple cells that express G-CSF range from endothelial cells to bone marrow stromal cells, while G-CSF receptors (G-CSFRs) are expressed on both hematopoietic and nonhematopoietic cells.^2^ G-CSF (Figure 1 in green) binds to G-CSFR (orange) in a 2:2 stoichiometry through crossover interactions between the G-CSFR Ig-like domain and the neighboring G-CSF. Residues from two sites on G-CSF are involved in receptor binding. These sites are the major site/site II (residues K16, G19, Q20, R22, K23, L108, D109, and D112) and the minor site/site III (residues Y39, L41, E46, V48, L49, S53, F144, and R147).^3^

Little is reported on major conformational changes in G-CSF that are significant to bioactivity or stability/aggregation. However, a G-CSF aggregation mechanism has been proposed in which a highly reactive and structurally perturbed monomer functions as an aggregation seed.^4^ This perturbation was suggested to be in loop AB of G-CSF by Raso et al., based on a change in intrinsic fluorescence and the location of tryptophan residue W58. The aggregation of G-CSF is potentially rate-limited by conformational stability^5,6^ consistent with such an aggregation-prone intermediate state. Peptide-level hydrogen–deuterium exchange mass spectrometry (HDX-MS) recently also confirmed the sensitivity of aggregation rates and thermal stability upon mutation or formulation, to changes the exchange rates of residues within loop AB, loop CD, and the beginning of loop BC.^7,8^ Identifying specific residues that instigate or are directly affected by significant structural changes like this, in response to mutations or thermal perturbations, could reveal important structural features and mechanisms that affect function and stability, and thus also guide future rational/semirational protein engineering.

Various biophysical methods can be used to assess the stability of proteins, including their conformational stability determined from changes in intrinsic tryptophan fluorescence during thermal or chemical denaturation. Colloidal stability can also be determined from aggregation onset temperatures, zeta-potentials and B_22_ values.^9−11^ Nevertheless, a blind spot exists when the stability of a protein is assessed simply by optical methods during thermal or chemical denaturation. They do not acquire any information regarding the changes that individual residues experience prior to and during denaturation or aggregation. They also ignore changes in the distribution of conformations within the native ensemble prior to denaturation, that may be functionally relevant. Thermal fluctuations of residues within the native ensemble are also considered to be an important aspect of the mechanisms that lead to aggregation behaviors.^12^ Furthermore, machine learning approaches have shown that the thermal dependence of fluorescence spectra under only native conditions, are sufficient to predict their subsequent melting temperatures,^13^ highlighting the underlying importance of native ensemble dynamics in defining the pathways to global conformational unfolding.

High-resolution insights on the residue-level dynamics over a range of native temperatures would provide valuable insights into key structural changes within the native ensemble that may be relevant to both function and the propensity to denature or aggregate. Observing residue-level NMR chemical shift and peak intensity changes over a range of temperatures from 295 to 323 K, we explore the changes that individual residues in G-CSF experience through the early stages of thermal denaturation prior to the global transition. The peak intensity of signals in NMR typically represent the population of a species in the solution, e.g., the more G-CSF molecules are in a particular conformation, the higher the observed peak intensities.^14,15^ Additionally, dynamics can influence peak intensity and there exists a plethora of NMR experiments to probe protein dynamics.^16−18^ Higher residue mobility decreases *R*_2_ (1/*T*_2_) relaxation rates and increases peak intensity.^19−21^ We attempted to identify dynamic residues in G-CSF, under conditions of pH 4.25 at which it is most stable for formulations, by collectively accounting for their change in microchemical environment and peak intensities.

Using this approach, we were able to resolve key events during the earlier thermal ramping toward the global transition temperature. This identified high-priority residues as potential targets for mutagenesis based on the significant changes they experience both locally and far in space. We also identified structural changes within loop AB that supports previous observations that this loop can conformationally rearrange to form an aggregation-prone state.^5,7,8^ Finally, we revealed subtle conformational changes in binding site III residues that may be significant in preorganizing the active site for receptor binding. Involvement of a key histidine residue suggests a pH dependence that may adapt G-CSF activity within the lower pH long bone marrow where it acts in vivo.^22^

## 2 Methods and Materials

### 2.1 Cell Culture

Minimal media were prepared for NMR using ^15^N-labeled (NH_4_)_2_SO_4_, PO_4_/NaCl (Na_2_HPO_4_, KH_2_PO_4_, NaCl), Na_2_SO_4_, EDTA trace elements (EDTA, CuCl_2_, ZnCl_2_, MnCl_2_, CoCl_2_, FeCl_3_, H_3_BO_3_), MgSO_4_, CaCl_2_, d-biotin, thiamine, and d-glucose. PO_4_/NaCl, Na_2_SO_4_, and EDTA trace elements were autoclaved at 120 °C for 20 min. The remaining components along with ampicillin (Amp), added to media at a final concentration of 1 mM, were filter sterilized with Millex-GP 0.2 μM, 33 mm, poly(ether sulfone) (PES) sterile syringe filters (Millipore, Hertfordshire, UK). A 100 mL seed culture of transformed *E. coli* BL21 (DE3) competent cells (New England BioLabs Inc., Ipswich, US) in minimal media/Amp was incubated overnight at 37 °C with shaking at 250 rpm. This seed culture was then transferred to a 2 L minimal media/Amp culture in a baffled flask and incubated (37 °C with shaking at 180 rpm) overnight. Expression was induced at an OD_600_ of 0.6, by spiking with sterile filtered isopropyl β-d-1-thiogalactopyranoside (IPTG) reaching a final concentration of 1 mM. The culture was left overnight at 37 °C with shaking at 180 rpm.

### 2.2 Primary Separations

Cells were harvested with centrifugation at 7080*g* for 20 min (4 °C) using an Avanti J-20 XPI (Beckman Coulter, Inc.). Cell pellets were then washed in 40 mL of 10 mM phosphate buffer saline (PBS) and centrifuged at 7728*g* for 30 min (4 °C) into ∼4 g pellets in 50 mL falcon tubes. To help with cell lysis, these pellets were stored at −20 °C. Each cell pellet was defrosted by leaving to stand for 30 min at room temperature (RT) and then resuspended in 40 mL of 10 mM PBS. Cell lysis was carried out by giving the resuspended pellets a single pass through a APV LAB40 high-pressure homogenizer at 1000 bar and storing them on ice. Cell lysis was also aided by adding sodium deoxycholate at 1 mg/mL and rolling at room temperature for 15 min. The high viscosity of the lysate from DNA release was reduced by addition of 20 μL of benzonase nuclease (25 U/mL; Merck Millipore) and rolling continued for 15 min. The lysate was centrifuged at 17,700*g*, 30 min, 4 °C (Avanti J20 XPI; Beckman Coulter, Inc., Fullerton, CA, USA) to pellet the GCSF inclusion bodies (IB). After removal of the supernatant, the IB pellet was washed twice to remove host cell impurities. In all steps, the pellets were resuspended in 160 mL of wash buffer using a homogenizer 850 (Fisher Scientific, UK) and repelleted via centrifugation at 17,700*g* for 30 min (4 °C). Wash A contained 50 mM Tris pH 8, 5 mM EDTA, and 2% Triton X-100 (w/v); wash B contained 50 mM Tris pH 8, 5 mM EDTA, and 1 M NaCl.

Pellet solubilization was achieved using a pH shift procedure, which included resuspension in 10 mL of 4 M urea and pH adjustment to pH 12 using strong NaOH, followed by rolling for 30 min at RT. Refold was achieved by diluting this solution dropwise by 20× into 1 M arginine·HCl buffer pH 8.25, followed by rolling for >12 h at RT. Refolding was quenched by pH adjustment to 4.25 using strong glacial acetic acid followed by rolling at RT for 2.5 h. The refold was clarified by centrifugation at 17,700*g*, 20 min, 4 °C (Avanti J-20 XPI; Beckman Coulter, Inc., Fullerton, CA, USA), and the supernatant was retained and concentrated to a final volume of 10 mL using an Amicon stirred cell (with 10 kDa, 29.7 mm diameter ultracentrifugal filter units; Merck Millipore). Concentration was continued with Amicon Ultra-15 10 kDa cutoff membrane centrifugal filters (Merck Millipore, Billerica, Massachusetts, USA) at 1389*g* and 4 °C.

### 2.3 Purification

The 10 mL concentrated sample was purified by size exclusion chromatography (SEC) on an ÄKTA Explorer (GE Healthcare Life Sciences, Germany) using a HiLoad 26/60 Superdex 200 prep grade column (GE Healthcare Life Sciences, Germany; 2.6 cm internal diameter; i.d., 60 cm bed height, 320 mL column volume; CV). A 10 mL injection loop was used to load the sample onto the column while elution was performed isocratically in 50 mM sodium acetate at pH 4.25 at 2.5 mL/min. Fractions with >0.1 mg/mL concentration were pooled and concentrated to a final stock concentration of 1.7 mg/mL (0.09 mM) using Amicon Ultra-15 10 kDa cutoff membrane centrifugal filters at 1890*g* and 4 °C.

### 2.4 NMR Spectroscopy

NMR spectroscopy was performed using a 700 MHz Bruker Avance NEO spectrometer fitted with a Bruker AEON refrigerated magnet and QCI-F cryoprobe. The ^15^N–^1^H HSQC spectroscopy experiments were performed using the *hsqcetfpf3gpsi* pulse sequence, while ^1^H NMR spectra were collected using the *zgesgp* pulse sequence. Spectra were recorded in the temperature range of 295–323 K at incremental steps of 2 K. To control for thermal drift of signals, 5 μL of 2,2,3,3-tetradeutero-3-trimethylsilylpropionic acid (TSP) was added to the protein sample.

### 2.5 Processing of Spectra and Further Analysis

Both ^1^H and ^15^N–^1^H HSQC experiments were processed in Topspin 4.0.8 (Bruker, Coventry UK). Signals from experiments at each temperature point were zeroed to the TSP signal. CcpNmr Analysis 2.4.2^23^ was then used for further analysis in order to calculate Δδ and peak intensity.

### 2.6 Rosetta

The Cartesian_ddg application within the Rosetta software suite was used to relax PDB 2D9Q. Gromacs was used to clean PDB 2D9Q with the “grep-v HOH” command and then renumbered so that residue 7 was residue 1. This renumbered PDB was then relaxed and the lowest energy PDB was taken for another relaxation step. The lowest energy PDB from the second relaxation step was then used as the relaxed structure in this study.

### 2.7 Calculating Solvent-Accessible Surface Area

Solvent-accessible surface area (SASA) was calculated using the lowest energy PDB from Rossetta on the online server ProtSA.^24^ A probe radius of 0.14 nm was used to perform this calculation.

### 2.8 APR Software

Consensus APRs were determined using AmylPred 2^25^ based on 10 APR scanning software, namely, AGGRESCAN, amyloidogenic pattern, average packing density, beta-strand contiguity, hexapeptide conf. energy, NetCSSP, Pafig, SecStr, TANGO, and WALTZ. The APR scanning software used in this study that was not part of this consensus is PASTA 2.0.^26^

### 2.9 Equations for NMR Observable Interpretations

The NMR observables δ and PI were scrutinized to give ∑Δδ, percentage change in PI, 90th percentiles of both observables and also residue correlation for both observables.

#### 2.9.1 ∑Δδ

Calculation of ∑Δδ is illustrated in Figure S.1. ∑Δδ at each temperature is the cumulative change in the microenvironment at that temperature, for example, ∑Δδ at 297 K = Δδ from 295 to 297 K and ∑Δδ at 301 K = (Δδ from 295 to 297 K) + (Δδ from 297 to 299 K) + (Δδ from 299 to 301 K).

#### 2.9.2 90th/95th Percentile for ∑Δδ

The 90th and 95th percentile for ∑Δδ was calculated at each temperature point along the thermal melt. The normal distribution of the ∑Δδ data set at each temperature was calculated and residues with a ∑Δδ above the 90th/95th percentile threshold of this distribution were considered to be in the 90th and 95th percentile, respectively. The normal distribution equation is given as
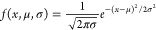
1where μ is the distribution mean, σ^2^ is the variance, and *x* is the independent variable.

#### 2.9.3 90th Percentile for PI

The same normal distribution equation was used to calculate the 90th percentiles for PI. Here, the normal distribution was calculated for all data points across the thermal melt and residues with a PI value above the 90th percentile threshold of this distribution were determined to be 90th percentile.

#### 2.9.4 Percentage Change

The percentage change in PI was calculated between the PI value at the start of the melt and maximum point of the melt for respective residues:
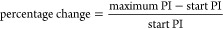
2

#### 2.9.5 Cross-Correlation for Δδ and PI

Spearman’s correlation (ρ) was used to calculate the correlation between residues. Coefficients were derived using δ and PI values at consecutive temperature points along the thermal melt. The Spearman’s equation used was as follows:
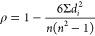
3where *d*_*i*_ is the difference between a pair of ranks and *n* is the number of observations.

## 3 Results and Discussion

### 3.1 Assigning G-CSF 2D ^15^N–^1^H HSQC Spectra at Different Temperatures

**Figure 2:**
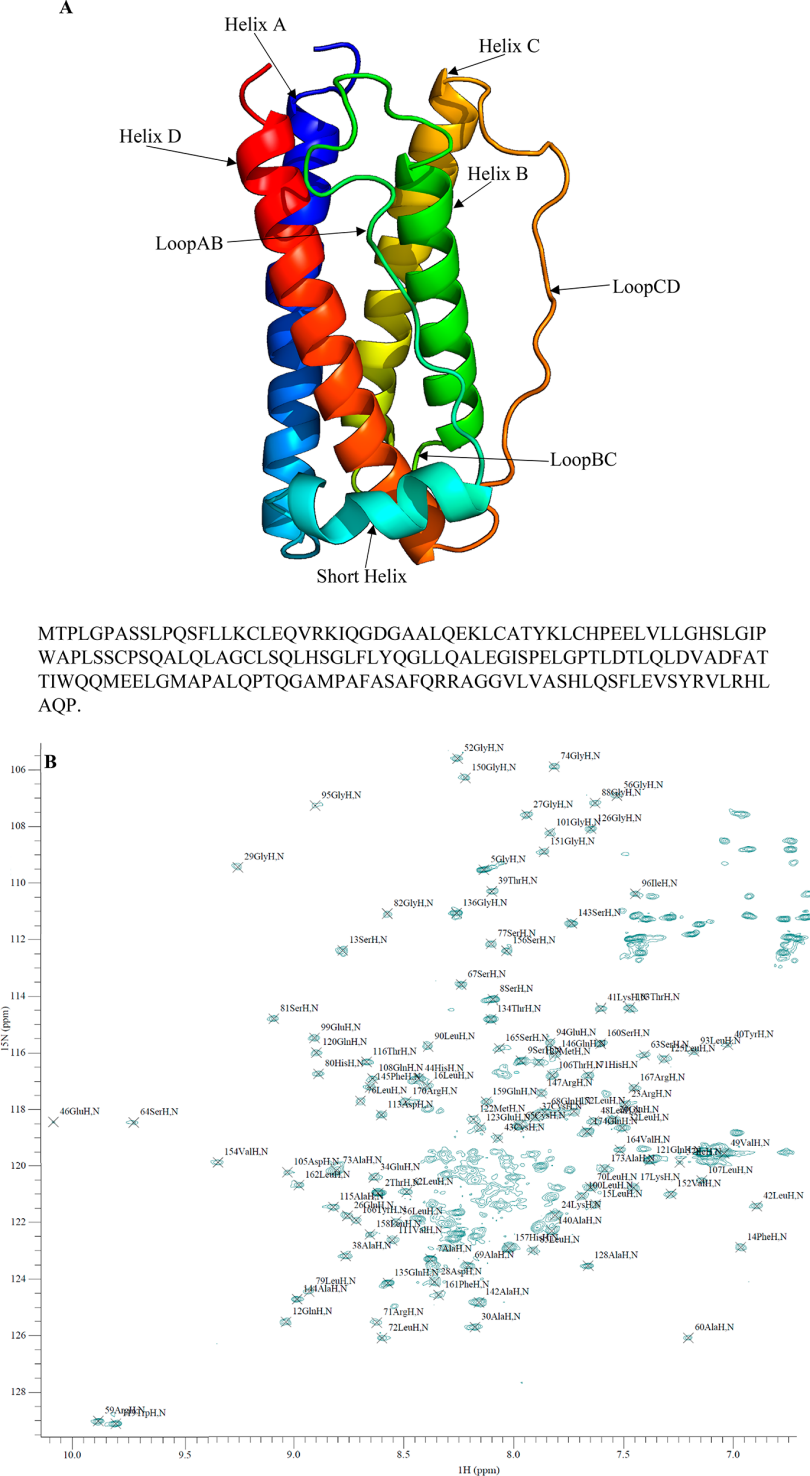
Assigned ^15^N–^1^H HSQC of G-CSF. (A) PDB 2D9Q of G-CSF and its amino acid sequence. (B) ^15^N–^1^H HSQC spectrum of 0.09 mM WT G-CSF (pH 4.25, 50 mM sodium acetate) collected at 303 K (all residue numbers are shifted by +1) and assigned in CcpNmr Analysis 2.4.2.^23^

From the 2D ^15^N–^1^H HSQC spectra of 0.09 mM wild-type G-CSF in 50 mM sodium acetate, pH 4.25 (Figure 2B), we were able to assign a maximum of 115 peaks out of the 160 assignable peaks published by Zink et al. using CcpNmr Analysis 2.4.2.^23,27^ We applied a thermal ramp from 295 to 323 K, which was just below the thermal melting transition temperature, and recorded spectra at every 2 K to track the movement of peaks by measuring the changes in their chemical shift positions (Δδ). This allowed us to monitor residue environmental changes (including partial unfolding events and conformational transitions) up until the point of global unfolding. The number of assignable peaks decreased from 115 at 295 K, to 106 at our final temperature of 323 K, where some peaks became coincident with others while others disappeared altogether.

**Figure 3:**
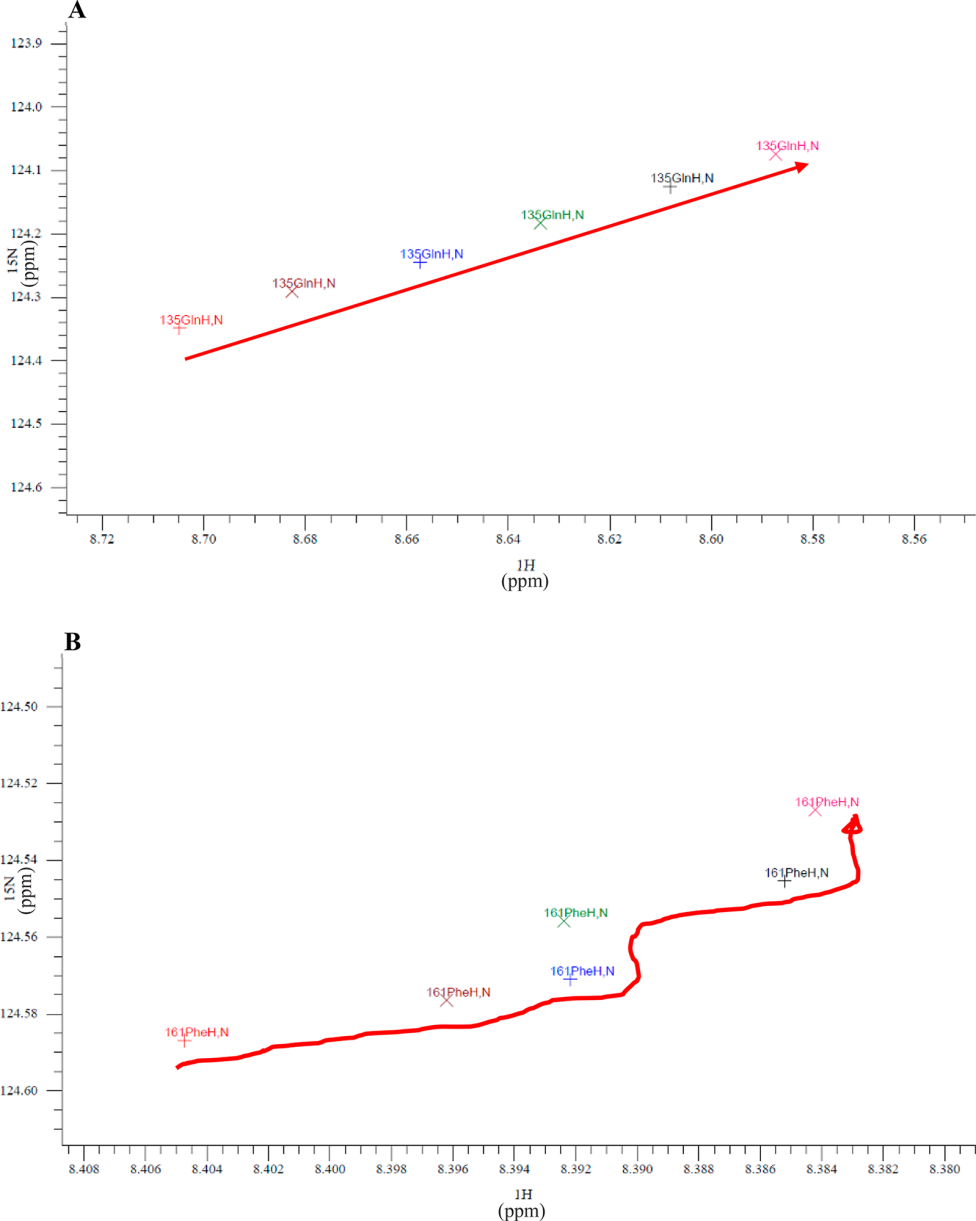
Residue peak migration over the thermal melt.

The chemical-shift distances traveled by peaks during the thermal melt were measured and the trajectories characterized as linear or nonlinear (defined in Table S.1). A typical example of a linear peak maxima trajectory during the thermal melt (295 K to 305 K) is shown in Figure 3A for residue Q134. In total, 68 residues had linear trajectories. By comparison, 44 residues had nonlinear peak trajectories over the thermal melt such as that in Figure 3B, which indicated a more complex pathway in their change in microenvironment, with intermediate conformations being populated. There is a concentration of some of the most nonlinear trajectories around the C-terminus of helix D (V163, R166, H170) and the proximal loop AB residues S62 and G73, the significance of which is later discussed. Some signals could not be assigned throughout the entire temperature range. For example, the peak from residue E45 disappeared at 303 K and above. The signals from other residues, namely, Q67, M126, E93, G87, and S155, were lost after appearing in the same position as a signal for another residue experiencing the same microchemical environment at that temperature. Residues with more than three temperature points missing were not included in further analysis.

Each cross in panels A and B represents the maxima from the peaks for residues Q134 and F160, respectively, from 295 K (represented with the black cross) to 305 K (represented with the pink cross). The red arrows indicate the trajectory of these maxima during the thermal melt.

### 3.2 Tracking the Cumulative Change in Residue Microchemical Environment

The peaks from different residues also moved at various rates and to varied extents, as shown with examples in Figure 3. To highlight this, a cumulative change in chemical shift (∑Δδ) was calculated for each residue as a function of temperature, starting from ∑Δδ = 0 Δδ at 295 K, as shown in Figure S.1.

The vast majority of residues had a linear “∑Δδ−temperature relationship” for the entire thermal ramp (Figure S.2), indicating a gradual rise in thermally induced mobility throughout the structure, but with no clear conformational changes in local structure. Their ∑Δδ values were normally distributed (Figure S.3), where the distribution broadened with increasing temperature, although 90% of residues remained with a total ∑Δδ of <0.2 Δppm even at 323 K. This indicates that the changes in microenvironment across the majority of the residues were mostly related to gradually increasing mobility in the native ensemble, with increasing temperature. On the other hand, a few key residues underwent significantly larger changes, surpassing the 90th percentile threshold of ∑Δδ (at 323 K) = 0.2 Δppm by at least 50%, indicative of residues with microenvironments much more susceptible to temperature than the majority.

**Figure 4:**
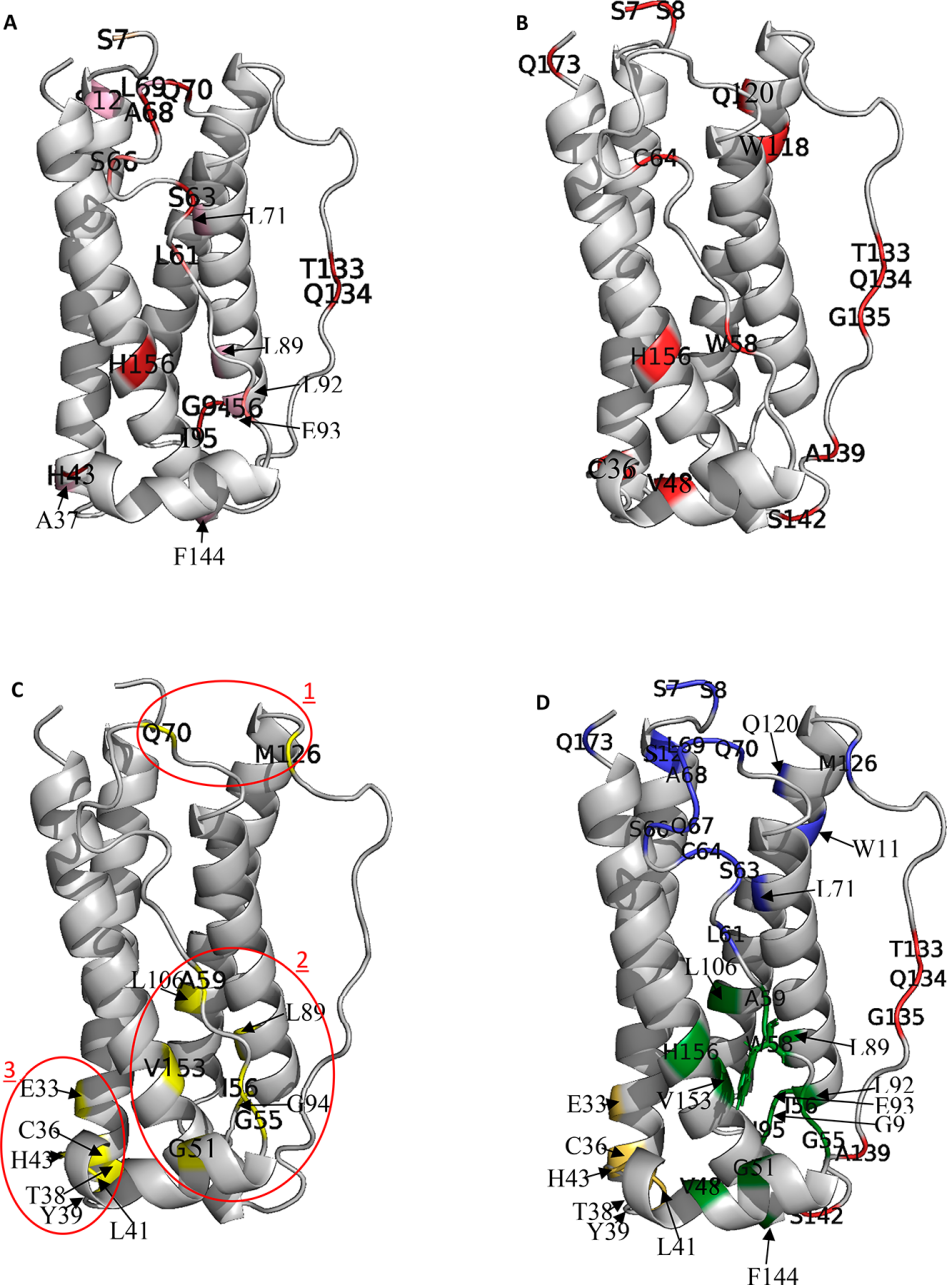
Mapping NMR Observables onto G-CSF. Each observable parameter is mapped onto the G-CSF crystal structure. (A) Residues in the 90th percentile of ∑Δδ. Residues that appeared in the 90th percentile for up to two of the 14 temperatures are colored pink, salmon residues appeared at 3–9 temperatures, and deep red residues appeared for at least 10 temperatures. In (B), all 90th percentile residues for PI (absolute change) are colored red. In (C), the 15 residues showing the highest percentage increase in PI over the temperature range are colored yellow. These form three subclusters, circled red. (D) Combines all residues in (A–C) to reveal four final structural clusters. Structural cluster 1 is blue, 2 is green, 3 is yellow, and 4 is red. Residue W58, assigned previously to observed hyper-fluorescence, is shown as sticks. PDB 2D9Q is missing its first 6 residues; therefore, S7 is highlighted in place of earlier residues.

Table 1A highlights residues ranked in the 90th percentile (colored yellow) and 95th percentile (colored green) according to their ∑Δδ at each temperature. Residue Q70 (the top gray line in Figure S.2) clearly ranked highest by ∑Δδ over the whole thermal melt except at 297 K. The relationship between residue-level ∑Δδ and location in the crystal structure of G-CSF (Protein Data Bank ID code 2D9Q)^3^ was also visualized by highlighting residues in the 90th percentile in Figure 4A. A more detailed color-mapping of ∑Δδ values for all residues, and at each temperature is available in the Supporting Information (Figure S.4). This shows the gradual increase in ∑Δδ for most residues as temperature increases but also highlights the positions of the residues that had stronger responses to temperature, which can be easily seen as early as 307 K.

**Table 1:**
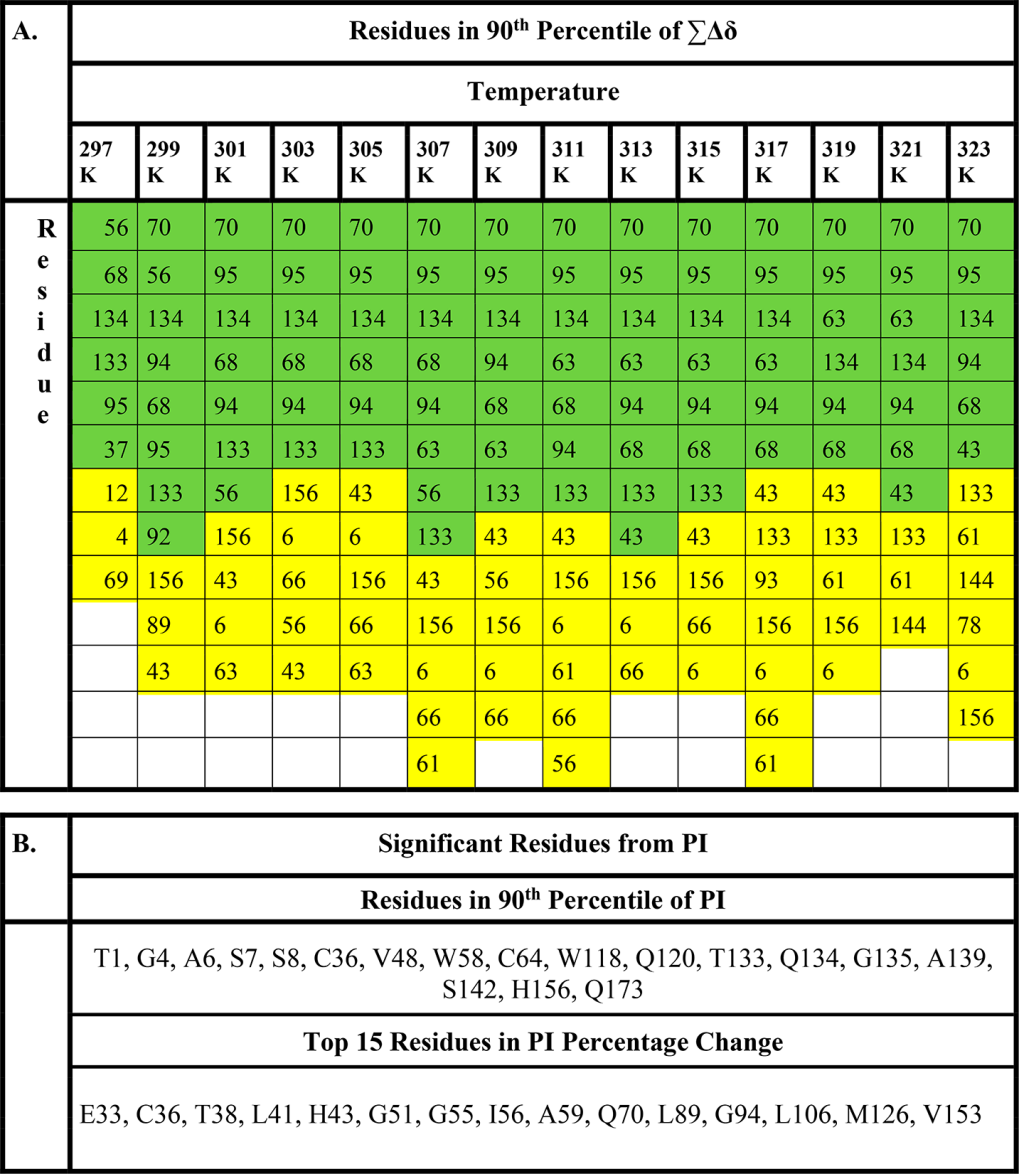
(A) Residues in the 90th Percentile (Highlighted Yellow) and 95th Percentile (Highlighted Green) of the ∑Δδ Normal Distribution at Each Temperature Point. (B) List of Significant Residues Determined from PI^a^

a Residues with a maximum PI value (typically around 305 K) that are in the 90th percentile are shown in the top half of the table. The top 15 residues with the highest percentage change in PI are shown in the bottom half.

Residues in the 90th percentile were mainly in loop AB, aside from residues H156, A37, L89, L78, F144, and E45, found in structural clusters formed from parts of helix A, B, D, and the short helix (Figure S.4). This suggests that there were three or four localized regions of structure susceptible to conformational change or partial unfolding at temperatures lower than for global unfolding. This will be discussed after further analysis below.

Interestingly, for a few residues (namely G55, I56, A59, S63, and F144), the ∑Δδ−temperature relationship was not entirely linear and a minor transition occurred at approximately 305/307 K, as visualized in Figures S.7 and S.8A. This transition represents a minor conformational rearrangement clustered within the first half of loop AB and its interactions with helix D, which is adjacent to the GCSF-R binding site III^3^. Notably, residue S63 climbs Table 1A rapidly at above 305 K as it experienced larger changes in its microenvironment during the localized conformational transition discussed above.

### 3.3 Variation of Residue Signal Peak Intensities with Temperature

**Figure 5:**
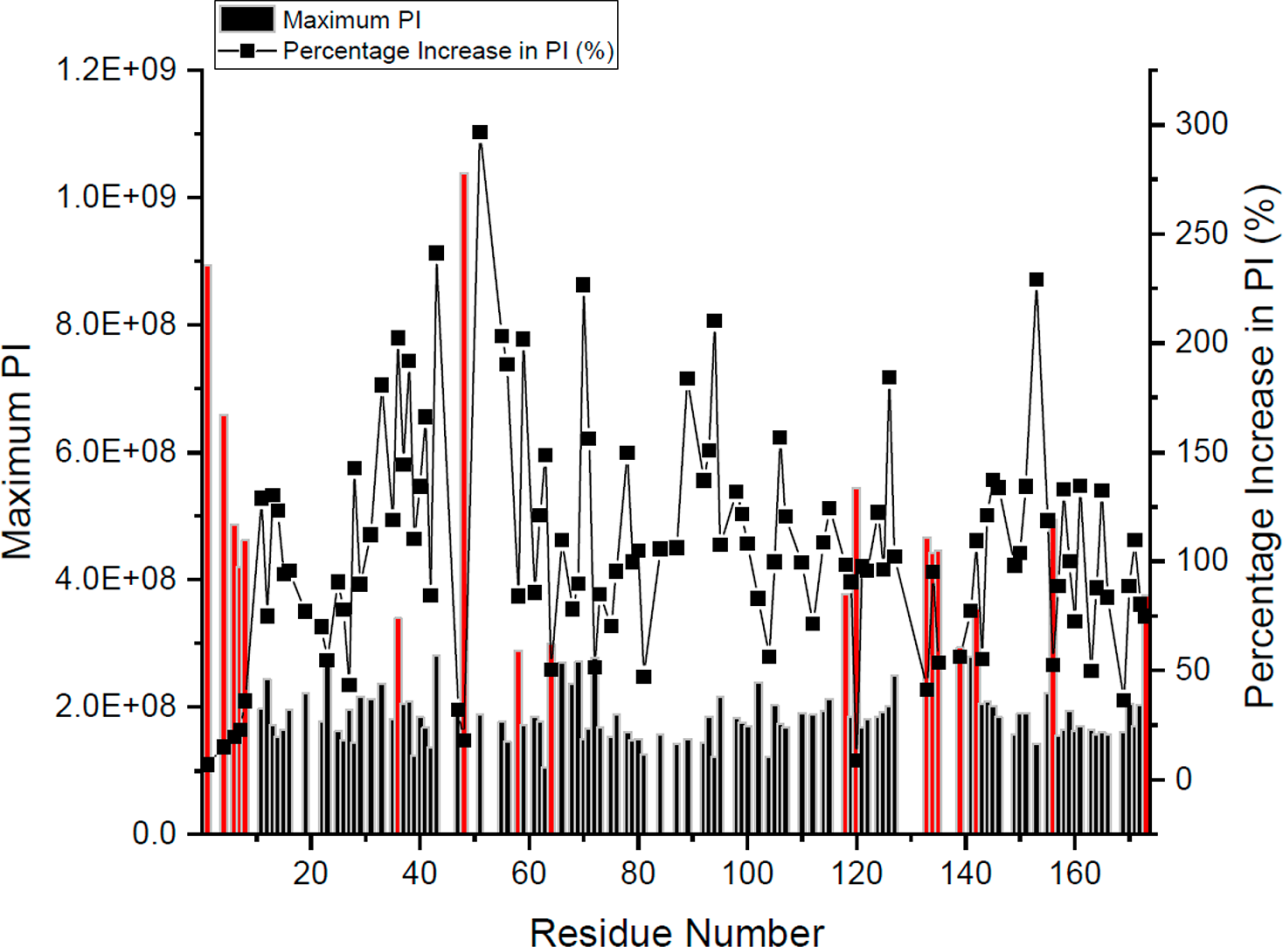
Maximum PI and percentage increase in PI. Maximum PI values for all residues (bars), with residues above the 90th percentile threshold for maximum PI highlighted red. The black line indicates percentage increase in PI over the melt.

Over the thermal ramp, we observed that the peak intensities (PI) for all residues, in general, increased with temperature up to ∼305 K and plateaued before decreasing at ∼313 K and above (Figure S.5) and could be fitted with a second order polynomial curve for all residues. This general trend relates to the gradual increase in dynamics, and hence PI, as the temperature increases, but then as the protein begins to unfold and aggregate, the PIs decrease, leading toward zero PI as the thermal denaturation midpoint is approached (at approximately 323 K). Residues T1 to S8, V48 and Q120 are an exception to this trend as they plateaued much earlier, perhaps signaling internal rearrangement or high dynamics while the protein was in a less energized state at lower temperatures. Residues in the 90th percentile of PI were determined as those passing the upper 10% threshold for the total distribution data for all PI (eq 1). Residues with a maximum PI above this threshold were classed as 90th percentile (indicated in Table 1B, Figure 4B, and Figure 5 with red bars). PI is strongly influenced by dynamics such that residues in the 90th percentile can be classed as relatively dynamic.

Given that both sample concentration and temperature can influence PI, we could, at least in part, be observing a general second order polynomial curve for PI due to the influence of these factors.^28,29^ The initial increase in PI could result from the rising temperature of the sample, which in turn increases bulk magnetization.^28^ Additionally, over a thermal melt, certain local conformations within the protein can become dominant. Consequently, this would cause the PI of these residues to increase over the melt, given that PI reflects the number of nuclei resonating at a given frequency (experiencing the same microchemical environment).^14^ Global increase in mobility would also cause this initially increase in PI. An interplay between all mentioned factors is equally likely. The decrease in peak intensity toward the end of the thermal melt could result from loss of sample through unfolding or aggregation as previously observed at around 321 K^7^ (323 K is where signals for the majority of residues are lost with NMR). Nevertheless, all residues may not follow these global trends and may hold key information to their role in global stability.

In addition to the maximum PI, we aimed to identify residues that underwent significant changes in dynamics during thermal denaturation. These large dynamic changes could result from local unfolding events, or conformational switching within the native ensemble with relevance to stability or function. The percentage increase in PI (eq 2) was calculated for all assigned residues (Figure 5). Therefore, unlike the 90th percentile for PI, percentage increase in PI refers to the largest change in dynamics experienced by a given residue. Residues that were highly dynamic already at low temperatures, such as at the N-terminus (T1 to S8) tended to give low (7–25%) increases in PI. However, some residues gave large increases in dynamics, such as G51 which experienced a 297% increase in PI. Many residues with a high percentage increase had low maximum PI values, and so had a low absolute change in PI. However, residues C36 and H43 have both high maximum PIs and a high percentage change, indicating significant changes in dynamics for these residues. The vast majority of the top 15 residues experiencing the highest percentage increase in PI (also highlighted in Table 1B and in yellow in Table S.3) were generally clustered at the receptor-binding end (site III) of the protein structure, forming the subclusters 2 and 3 shown in (Figure 4C). This is also particularly emphasized in the split halfway through loop AB in which the N-terminal residues had large percentage increases in PI, compared to the C-terminal half of loop AB which gave high maximum PI values.

There was considerable overlap between the residues in the 90th percentiles of ∑Δδ, PI, and %PI (Table 1) and hence also for their structural locations (Figure 4A–C). Figure 4D combines the residues highlighted by each measure, and clearly shows that they form four structural clusters. The first is formed along the C-terminal half of loop AB (residues L61, S63, C64, S66, A68, L69, Q70, and L78), the C-terminus (Q173), the N-terminus (T1, G4, A6, S7, S8, and S12), and the beginning of loop CD/end of helix C (W118, Q120, and M126). The second structural cluster spans the N-terminal half of loop AB (G55, I56, W58, and A59) with some interacting residues from the short helix (V48 and G51), helix D (F144, V153, and H156), helix C residue L106, and loop CD residues (L89 and L92-I95). From the residues involved, this structural cluster appears to form a large hydrophobic core in which residues also have a low solvent accessibility (Figure S.9A). Therefore, given that an increase in solvent accessibility can increase transfer of magnetization to solvent (thereby increasing PI), structural cluster 2 could be experiencing an expanding motion, making it more solvent-accessible.

The third structural cluster resides in GCSF-R binding site III, with helix A and nearby short helix residues (E33, C36, A37, T38, L41, and H43). Finally, the fourth structural cluster is centered on loop CD residues T133, Q134, G135, A139, and S142 at the end of loop CD, near to the short helix. Clearly, these structural clusters overlap in some regions.

As discussed above, the large changes in microenvironment or dynamics for these residues in localized structural clusters, indicates localized conformational changes or partial unfolding, at low temperature (from 305 K) prior to any global unfolding or aggregation which begins (>1% unfolded) at approximately 320 K^6^. The focus around loop AB is consistent with previous work implicating this region in a conformational shift to form an aggregation-prone G-CSF intermediate.^5,30^ More recent HDX-MS studies on G-CSF formulations containing mannitol, phenylalanine, or sucrose^7^ and on single mutant variants of G-CSF^8^ have confirmed the role of loop AB. In addition, they revealed changes in dynamics within the short helix, loop CD and part of helix D, that correlated with aggregation propensity and the thermal melting temperatures (*T*_m_). Our NMR data, showing structural clusters 1, 2, and 3 encompassing loop AB, and structural cluster 4 within loop CD, fully supports a conformational change localized in these same regions, that is promoted through the moderate temperature increase to 307 K. The largest aggregation-prone region (APR), identified by the consensus method employed in Figure S.11, spans helix D. Conformational changes around loop AB have a strong potential to expose this APR. Given the nonlinear trajectory for residues clustered around the N-terminus of helix D (V163, R166, and H170) and proximal C-terminus of loop AB (S62 and G73), conformational changes in these regions could result from multiple states being occupied in loop AB before helix D is exposed.

A notable feature of the structural clusters identified by NMR, is that none of them are directly involved in the major binding site (site II) containing residues K16, G19, Q20, R22, K23, L108, D109, and D112. Thus, the structural rearrangements identified as the temperature is increased would not necessarily affect the integrity and function of binding site II. However, the minor binding site (site III) appears to be directly impacted, suggesting that this site can be conformationally switched on or off. Indeed, the significant distortion of loop AB may be necessary to elicit a conformational change in binding site III for receptor interaction, as eluded to in Figures S.8 and S.10. It appears, therefore, that the structural change identified as making G-CSF more aggregation-prone in vitro, is the same or similar to the structural change observed at 307 K in vitro. It is also very possible that the higher temperature structure is the functionally relevant state in vivo at 37 °C (310 K), although the difference in pH from our work at pH 4.25, and physiological pH of 6.7–6.9 in long bone marrow, would also likely have an influence.^22^

### 3.4 Probing Correlations in Δδ and PI

**Figure 6:**
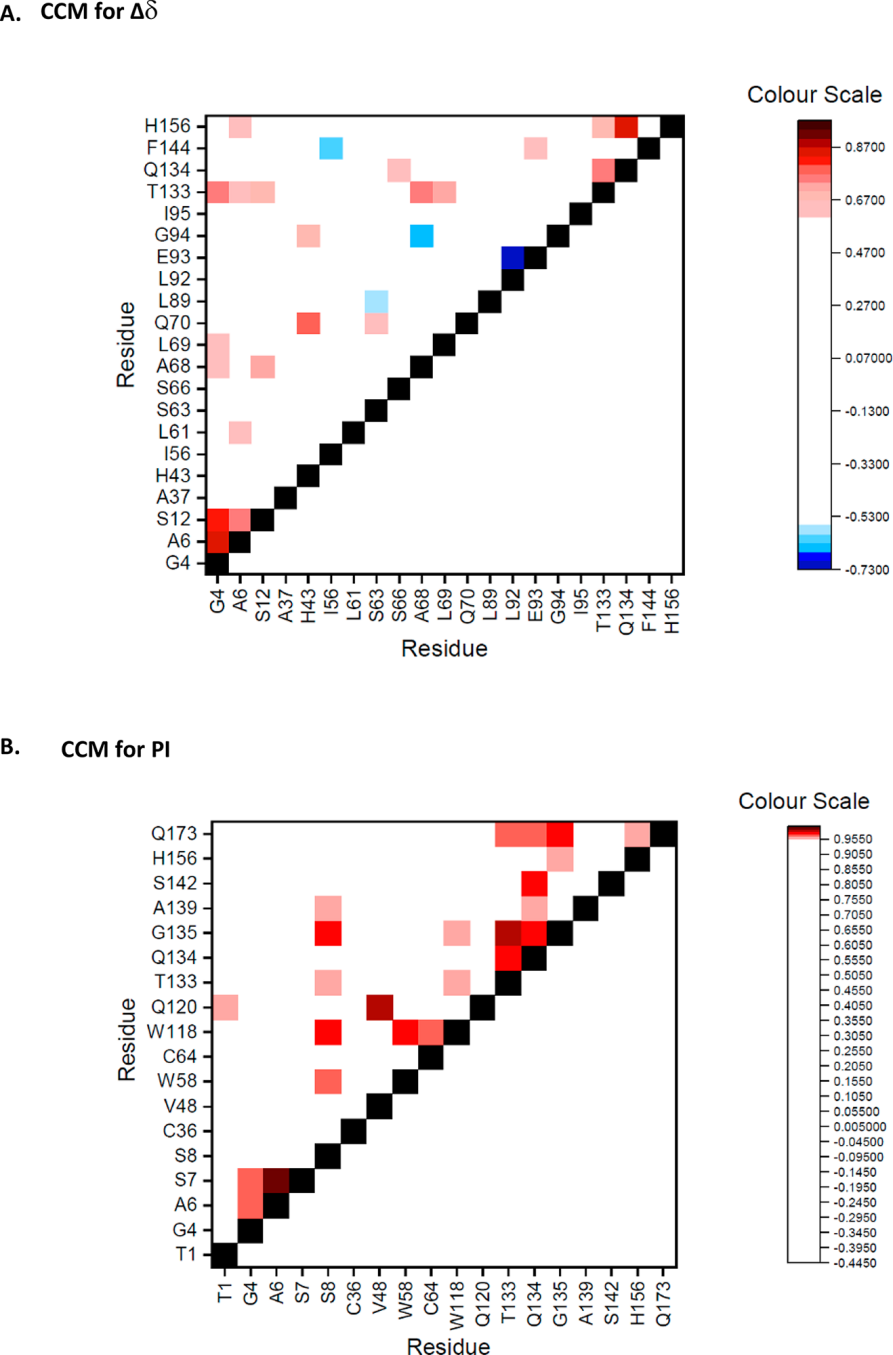
Cross-correlation matrices (CCM) for (A) Δδ and (B) PI. Spearman’s correlation coefficient values are color coded. In (A), shades from red to black represent positive correlation (above 0.6) and shades from blue to purple represent negative correlation (below −0.6). A coloring gate of white was used between −0.6 and 0.6 to reduce noise. In (B), shades from red to black represent positive correlation (above 0.95) and shades of blue represent negative correlation (below −0.95). A coloring gate of white was used between −0.95 and 0.95 to reduce noise.

Although ∑Δδ, PI, and %PI indicated which residues were undergoing the most change under “native” conditions prior to the global thermal melt, this did not reveal how the movements in each residue related to the others, beyond simply colocating them in structural clusters. Correlation analysis between residues could determine whether the changes in residues or the structural clusters are directly coupled during the thermal denaturation. Figure 6A shows a cross-correlation matrix (CCM) for the temperature-dependent Δδ of all residues in the ∑Δδ 90th percentile over the entire temperature range studied. Figure 6B shows a similar CCM for residues in the 90th percentile of PI, but correlating across all of their PI values at respective temperatures.

Correlations were determined in each case using the Spearman’s correlation coefficient (eq 3). For Δδ, each coefficient value was calculated between a pair of residues’ Δδ values from consecutive temperature points over the thermal melt. Using the example of residue 90 in Figure S.1, residue Q90s D_1_, D_2_, D_3_, etc. would be correlated with residue E33s D_1_, D_2_, D_3_, etc. In the color scale for Figure 6A, the red shades represent positive correlations above 0.6, and the blue shades represent negative (anti-) correlations of less than −0.6. A white color gate is used between 0.6 and −0.6 to remove noise or correlations of low confidence. The diagonal black line occurs through the matrix where complete correlation occurs between the same residues. Most space in this matrix is white, indicating that most residues do not elicit strong correlations between their temperature-dependent fluctuations in Δδ. However, there are some clear regions of strong positive or negative correlation.

For PI, positive correlation values were generally higher than those for Δδ. Therefore, a higher color gate (−0.95 to 0.95) was needed to reduce noise. Negative correlation did not occur below a value of −0.95

Interestingly, residues within loop AB itself did not tend to correlate strongly with each other in either matrix, suggesting that the overall change in conformation across the loop was not highly concerted or cooperative, but was probably more progressive with temperature. This is also reflected in the C-terminal end of loop AB having more 90th percentile PI residues (highly dynamic), but the N-terminal end having more 90th percentile %PI residues (large change in dynamics).

By contrast, several key clusters with strong correlations were observed in the two matrices. Some were formed within their local sequence (close to diagonals on matrices), such as at the N-terminus (G4, A6, S7, and S12), within loop CD (T133, Q134, and G135), and within loop BC (L92 and E93). However, the two regions in the N-terminus and loop CD are also strongly correlated with each other despite being spatially distant (30.1 Å apart). A key linker between these regions appears to be residue W118 in helix C, which sits between them spatially, and has strong correlations with S8, T133, and G135 in PI.

The loop CD cluster (T133, Q134, and G135) was also correlated to regions of loop AB (S66, A68, and L69) close to the N-terminal end of loop CD and to H156 at the other end of loop CD, indicating increased dynamics in the center of loop CD resulting from modified interactions with the ends of that loop. As the C-terminal end of loop AB (A68 and L69) was also strongly correlated in Δδ to N-terminal residues (G4 and S12), this provides another structural link that could mediate the correlation between the N-terminus and loop CD. Thus, overall, the structural changes in the N-terminus, the C-terminal end of loop AB, the center of loop CD and residue W118, appear to become modified in a concerted manner. The changes in the N-terminal end of loop AB is not in concert with this but instead undergoes its own nonlinear transition at ∼305 K as seen for residues G55, I56, A59, and S63 (Table S.2B and Figure S.8).

A few other individual residues show strong correlations, without forming clusters with local sequence. As such, while they may indicate coupled loss of interactions, their spatial separations and occurrence as individual residues suggests that they are more likely to be coincidentally undergoing similar changes in microenvironment.

### 3.5 Characteristics of Structural Cluster 3 Reveals a Potential “Switch Mechanism”

**Figure 7:**
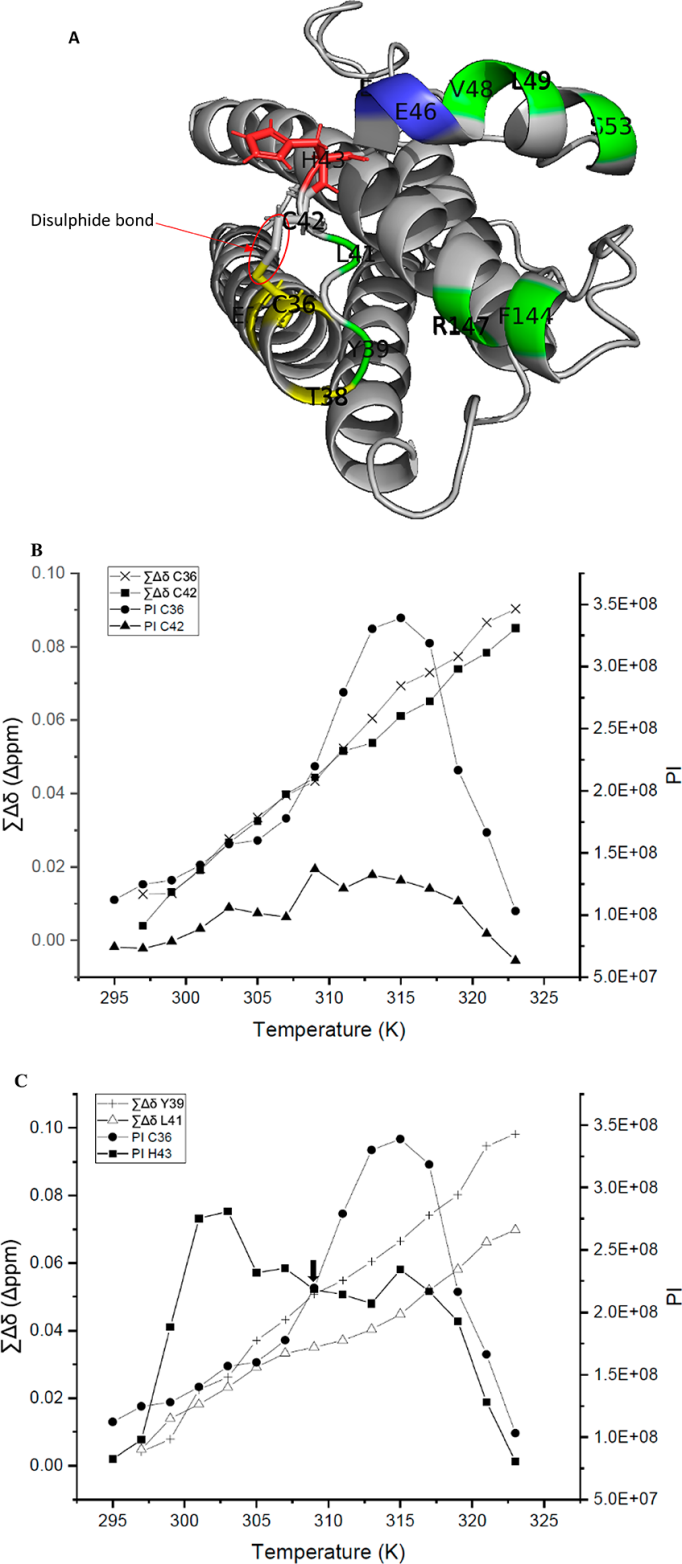
“Switch” mechanism. (A) Causal and beneficiary residues in the “switch” mechanism. The disulfide bond between C36 and C42 is indicated in the red circle. Structural cluster 3 residues are yellow, H43 is red, E45 and E46 are blue, and residues in GCSF-R binding site III are green. (B,C) Comparison of PI and ∑Δδ for C36, C42, Y39, and L41. A black arrow represents the point of physiological temperature (∼309 K) in (C).

Residue H43 displayed a notably high ∑Δδ, maximum PI, and percentage change in PI (Table 1 and Figure 5) and appears in structural cluster 3 (Figure 4D). From this cluster, residue C36 is disulfide bonded to C42, and while both residues showed a similar PI profile up to 307 K, there was a clear inflection point for C36, where its PI increased more rapidly at above 307 K and peaked at 315 K (Figure 7B). PI for residue C42, on the other hand, slightly increased at 307 K as well but then decreased and stayed low after this. Similarly, ∑Δδ for residues C36 and C42 were similar up until 311 K but then clearly differentiated at the same temperature as the large peak in PI for residue C36.

Residue H43 is adjacent to the disulfide bonded residue C42 (Figure 7A) and is also proximal to a very negatively charged area composed of E45 and E46 (highlighted blue). P44 is positioned such that it angles the side chain of H43 toward these negatively charged residues. H43 also has the second highest percentage increase in PI (241%), visible as a distinct peak in PI at 303 K occurring immediately before the peak observed in PI for C36 in Figure 7C. The physiological temperature of ∼309 K (indicated with a black arrow) occurs right at the transition point just after the decrease in PI for H43 and just before the peak in PI for C36. Therefore, H43 is well-placed to instigate a “switch mechanism”, with attraction toward E45 and E46 placing a strain on the disulfide bond between C42 and C36, due to the pulling of the short loop containing H43. A significant PI increase was experienced by C36, and much less so for C42, because it is part of a structured α-helix with more restricted movement. The significant PI increase for C36, and decrease for H43 suggests a shift toward a new conformer with increased dynamics for C36, potentially also including breaking of the disulfide, and with decreased dynamics for H43 as it forms stronger interactions with E45 and E46. Of note, although processing of NMR observables for E45 is not shown due to more than three missing temperature points, the PI for E45 decreased simultaneously with the increase in PI for H43. This could signify increased conformational restraint on E45 as H43 interacts more with it.

Given that H43 is part of an unstructured loop between helix A and the neighboring short helix region, the proposed “switch mechanism” would likely expose the loop region that also contains residues L41 and Y39 (highlighted green in Figure 7A). ∑Δδ of Y39 and L41 sharply increases during H43’s PI maximum, with a slight slowing to that increase, while C36 reached its PI maximum (Figure 7C). Both Y39 and L41 form part of the minor G-CSF receptor (GCSF-R) binding site III, highlighted green in Figure 7A.^3^ Furthermore, they are among the most buried residues in both active sites for G-CSF, displaying a solvent-accessible surface area (SASA) of 0.640 and 0.099 nm^2^, respectively (in Table 2 and Figure S.9A) as determined using the online server ProtSA.^24^ That makes L41 the most buried residue in both of G-CSF active sites and Y39 the fifth most buried (Table 2). Hence, exposure of these residues by a “switch mechanism” would have a significant impact on bioactivity. These observations were found to be highly reproducible for WT G-CSF in the same buffer with a range of different excipients added (Figure S.12).

**Table 2:** SASA of All of These Residues in Binding Site III

residue	SASA (nm^2^)
Leu-41	0.099
Gln-20	0.160
Asp-109	0.161
Val-48	0.296
Tyr-39	0.640
Arg-147	0.662
Asp-112	0.779
Ser-53	0.937
Lys-16	1.049
Glu-19	1.064
Arg-22	1.082
Lys-23	1.128
Phe-144	1.136
Glu-46	1.140
Leu-49	1.203
Leu-108	1.223

### 3.6 In Silico Structure Relaxation Supports NMR and a Proposed “Switch Mechanism”

**Figure 8:**
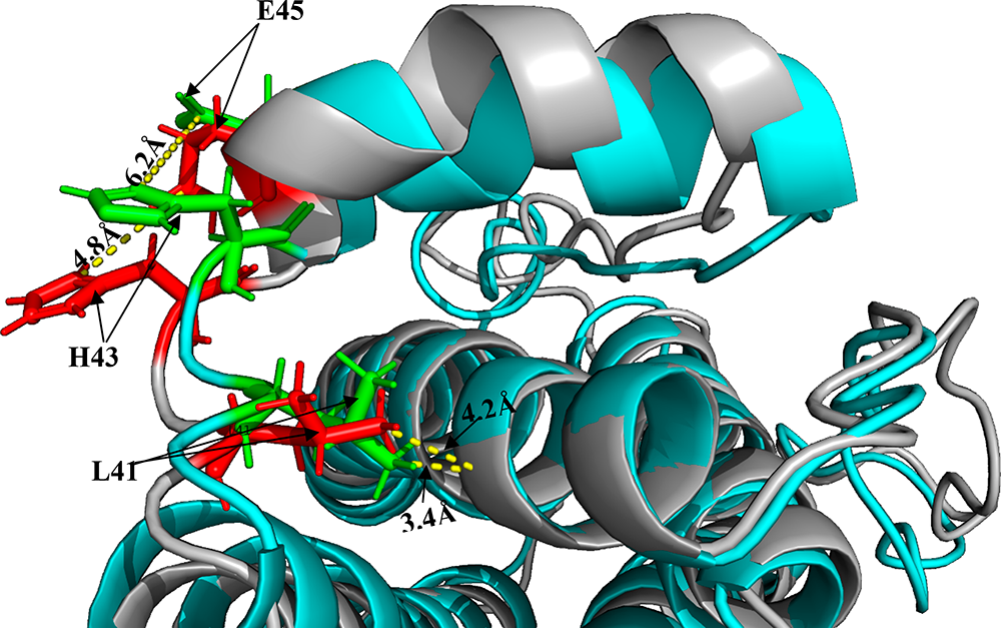
PDB 2D9Q vs relaxed structure. PDB 2D9Q relaxed structure in Rosetta Cartesian_ddg (cyan) overlaid on an unrelaxed PDB 2D9Q (gray). H43 and E45 are highlighted green in the relaxed structure, and H43 and E45 are highlighted red in the unrelaxed structure. Distance between both residues is indicated in the relaxed and unrelaxed as 6.2 and 4.8 Å, respectively. L41 is highlighted in the same manner as this with its distance from the neighboring α-helix backbone being 3.4 Å in the relaxed structure and 4.2 Å in the unrelaxed structure.

To further understand the observed changes in ∑Δδ and PI, the PDB 2D9Q structure of the receptor-bound G-CSF was relaxed using the online server Rosetta in the absence of the receptor. This would allow residues that are thermodynamically constrained in the bioactive receptor-bound state, to relax into conformations more favored in the unbound state, and that could then be compared to the large changes observed for ∑Δδ and PI. While this relaxation approach is not tailored for specific buffer compositions or pH, it does at least provide some insights into structurally stable forms in the complexed and noncomplexed state. The relaxed structure (colored cyan) is compared with the unrelaxed PDB 2D9Q structure (colored silver) in Figure 8.

Structural cluster 2 is the second largest of the four (Figure 4C). All of these residues, aside from G94 and A59, form a hydrophobic pocket with their side chains in close proximity (within 4.7 Å) to each other (Figure S.10B). The H atom of G94 and side chain of A59 face toward this hydrophobic region and both residues are highly buried with a SASA of 0.106 and 0.246 nm^2^, respectively. Structural cluster 2 is also very close to V48, which has the highest maximum PI by a large margin (Figure 4/5). When comparing relaxed G-CSF (cyan) with unrelaxed (silver) in Figure 8, the short helix next to the structural cluster 2 hydrophobic region clearly moves outward in the unrelaxed structure (Figure S.10B). V48 appears to lead this outward movement of the short helix. In the relaxed structure the short helix is fairly straight, whereas in the unrelaxed structure, it is curved with V48 at the apex. This structural change is not picked up by NMR as a significant change in the microenvironment of V48 because it is already solvent exposed (Table 1 and Figure S.9A) and so already highly dynamic. The large cluster of hydrophobic residues in structural cluster 2 that experience a significant percentage increase in PI over the thermal ramp suggests that they become more mobile as the hydrophobic core unpacks, alongside a movement in position of the short helix. The intensity of the signal can in part depend on the ability of side chains to transfer magnetization to the solution. Therefore, expanding of the hydrophobic core region could also cause these residues to become more solvent exposed and significantly increase their signal. Moreover, residues G55, I56, and G149 experience extremely nonlinear peak trajectories (Table 1 and Figure S.6) and are within (and close to) structural cluster 2, suggesting that this N-terminal loop AB region adopts multiple conformations as it expands.

H43 was in the 95th percentile of ∑Δδ, had the second highest percentage increase in PI (Table 1 and Table S.3) and was close to being in the 90th percentile for PI (Figure S.9A). This suggested that it underwent a significant environmental change affecting its dynamics as the temperature increased. Given that this residue would be positively charged under our experimental conditions, it would also be attracted toward the nearby negatively charged E45 and E46 residues (Figures 7A and 8). Figure 8 highlights residues H43, E45, and L41 in green (relaxed) and red (unrelaxed). H43 can be seen to move further from E45 upon relaxation, shifting from 4.8 Å apart in the receptor-bound state to 6.2 Å apart in the relaxed unbound structure. Moreover, the backbone of the loop containing L41 moved slightly, while the side chain became more tightly packed onto the helix D backbone in the relaxed structure, compared to a more solvent exposed position in the unrelaxed structure, undertaking a 0.8 Å shift in position. Overall, the changes observed upon relaxation into the unbound structure appear to correspond with our NMR-observed transitions in reverse, and so from higher to lower temperature structures. This places G-CSF into a more active conformation at above 309 K (36 °C) in vivo, although it should be stressed that our studies were at pH 4.25.

This “switch” involving H43 could be significant to bioactivity because it is part of the same short unstructured loop as L41 and Y39. Both of these residues are part of the GCSF-R binding site and are buried (Table 2).^3^ Hence, the aforementioned movement by H43 could pull L41 and Y39 out into solution so that they are more exposed to allow receptor binding. Supporting this is the sharp change in the microenvironment of L41 and Y39 at the same time as the increase in H43’s PI and our relaxed structure comparisons (Figures 7C and 8). This “switch” appears to come at a cost to C36. The large increase in C36’s PI beginning at ∼307 K places it in the 90th percentile of PI. However, at physiological temperature (309 K), G-CSF would not experience this possible strain on C36 but would experience the benefit of the H43 “switch” (Figure 7C).

G-CSF is most stable at pH <7, ideally pH 4. Suggested contributions to this characteristic range from high colloidal stability to stronger cation-π interactions between residues W58 and H156 (both of which are very dynamic according to our study) at low pH.^30,31^ Furthermore, while the pH of bone marrow (where GCSF-R is present) is not well studied, some studies suggest it to be slightly acidic.^22,32^ The lower pH would therefore increase the attraction of H43 toward E45/E46, thus supporting a potential “switch mechanism” controlled by H43 as seen when the unrelaxed and relaxed G-CSF structures are overlaid (Figure 8). Bone marrow is also proposed to be more reducing than the intravascular environment, which could lead to a larger population of the reduced disulfide bond near H43, giving it more freedom to make the “switch”.^33,34^ However, it should again be noted that our work at pH 4.25 is relevant to G-CSF formulations, but is not an exact comparison to physiological conditions.

Although V48 is part of the active site, its importance to bioactivity could be more than just binding to the receptor. Its dynamic nature could facilitate the expansion of binding site III (Figures 7A and S.10B), which could aid with the complementarity of this binding site to the receptor.^3^ Additionally, it could act in combination with the H43 “switch” to help expose L41 and Y39.

## 4 Conclusion

NMR was able to assign and track the mobility of G-CSF residues across a range of temperatures prior to any global unfolding. Our findings were highly consistent with previous observations of the influence of loop AB and surrounding structure on G-CSF stability and aggregation propensity. We found that physiological temperature induced structural changes in a local structural cluster around loop AB that corresponded to regions previously linked to the formation of an aggregation-prone state. Furthermore, the same structural changes were important for “switching on” of bioactivity through remodelling of the receptor binding site III. The implication is that while the use of formulation approaches remains highly suitable for stabilizing against the aggregation-inducing conformational change in a product vial or syringe, the use of protein engineering strategies to stabilize against the same structural changes may have knock-on functional effects in vivo. Our findings also provide further insight into why the *T*_m_ values are often a poor predictor of aggregation kinetics when stored at lower temperatures.^6^ The thermally induced conformational switch at 307–310 K (34–37 °C) would mean that the global unfolding measurement of *T*_m_ is made from a different native state than the one present at the lower temperatures used for drug product storage. Finally, the remodelling of loop AB and binding site III involves a critical change in the position of residue H43, which points to a likely pH-sensitivity, including the reason why G-CSF is more stable at pH 4.25 in vitro than at physiological pH. It is also possible that the pH sensitivity is an important feature in G-CSF activation in long bone marrow which has a slightly acidic pH.

## Supporting Information Available

Derivation of ∑Δδ; temperature dependence of ∑Δδ for all assigned residues; normal distribution of ∑Δδ at each temperature; mapping ∑Δδ to G-CSF structure; PI dependence on temperature; linearity of peak trajectories Δδ; ∑Δδ for 90th percentile residues; *R*^2^ values for linearity of ∑Δδ vs temperature for each residue; temperatures at which residues become nonlinear in their trajectories for ∑Δδ; schematic temperature line; percentage change in PI; maximum PI vs SASA; proximity of residue S8 to Q70; identifying APRs; comparison of the “switch mechanism” in different buffers (PDF)

## Definitions

∑Δδ: cumulative change in chemical shift
PI: peak intensity
90th percentile of PI: the upper 10% threshold for the total distribution of PI data.
percentage increase in PI: the percentage increase in PI (typically between 295 and 305 K)
linear/nonlinear “peak trajectory”: linearity of peak maxima movement across the HSQC spectra
linear/nonlinear “∑Δδ−temperature relationship”: linearity of ∑Δδ vs temperature lines
subcluster: cluster of residues from the top 15 residues for percentage increase in PI
structural cluster: cluster of residues considered significant from combined ∑Δδ, PI and percentage increase in PI
