# Supplementary material for: NMR Reveals Functionally Relevant Thermally Induced Structural Changes within the Native Ensemble of G-CSF
Source: Mol Pharm. 2022 Aug 10;19(9):3242–55. doi: 10.1021/acs.molpharmaceut.2c00398 (PMC9449972; doi:10.1021/acs.molpharmaceut.2c00398)
Supplement: Supplementary file 1 — mp2c00398_si_001.pdf [file mp2c00398_si_001.pdf]

Supplementary Information

NMR Reveals Functionally Relevant Thermally-induced Structural Changes within the Native Ensemble of G-CSF

Mark-Adam W. Kellerman[1*], Teresa Almeida[2], Timothy R. Rudd[2,3], Paul Matejtschuk[2], Paul A. Dalby[1]**

[1]Department of Biochemical Engineering, University College London, Gower Street, London, WC1E 6BT

[2]National Institute for Biological Standards and Control (NIBSC), Medicines & Healthcare products Regulatory Agency, Blanche Lane, South Mimms, Potters Bar, Hertfordshire, EN6 3QG

[3]Department of Biochemistry and Systems Biology, Institute of Systems, Molecular and Integrative Biology, University of Liverpool, Liverpool, L69 7BE

***Email: Mark-adam.kellerman.18@ucl.ac.uk**
****Email: p.dalby@ucl.ac.uk**

Using δ to Calculate ∑Δδ, Peak Linearity, Slope and the Normal Distribution of ∑Δδ

S.1 illustrates the concept of calculating ∑Δδ where, for example, the ∑Δδ at 297 K = Δδ from 295 K to 297 K, and the ∑Δδ at 301 K = (Δδ from 295 K to 297 K) + (Δδ from 297 K to 299 K) + (Δδ from 299 K to 301 K). The example in this figure demonstrates how the ∑Δδ for residue Q90 at 301 K is equal to the sum of the Δδ distances travelled between the peaks for residue Q90 at previous temperatures.

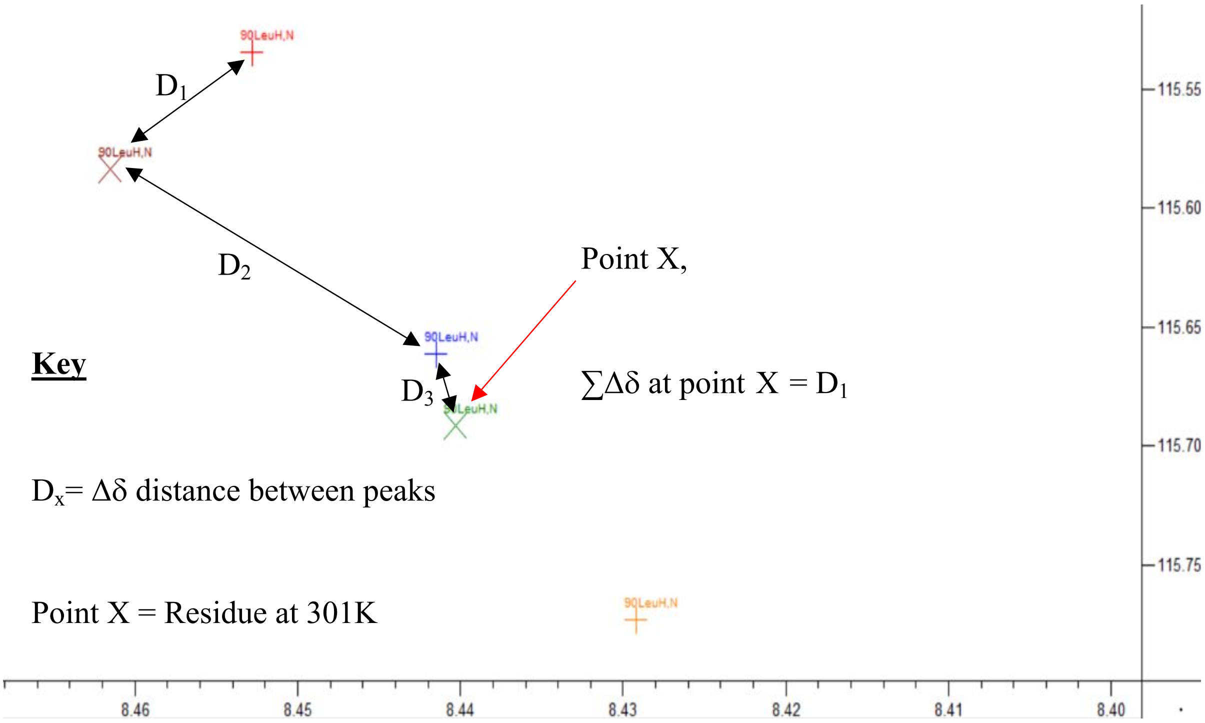

Figure S.1. Deriving ∑Δδ.

The movement of the peak maxima for residue L89 is given here by the different coloured crosses, with red representing its position at 295 K and orange at 305 K. D_x gives the Δδ distance between peaks at consecutive temperatures. ∑Δδ at point X, i.e. the total Δδ distance travelled by residue L89 at 301 K, is given as $D_1 + D_2 + D_3$.

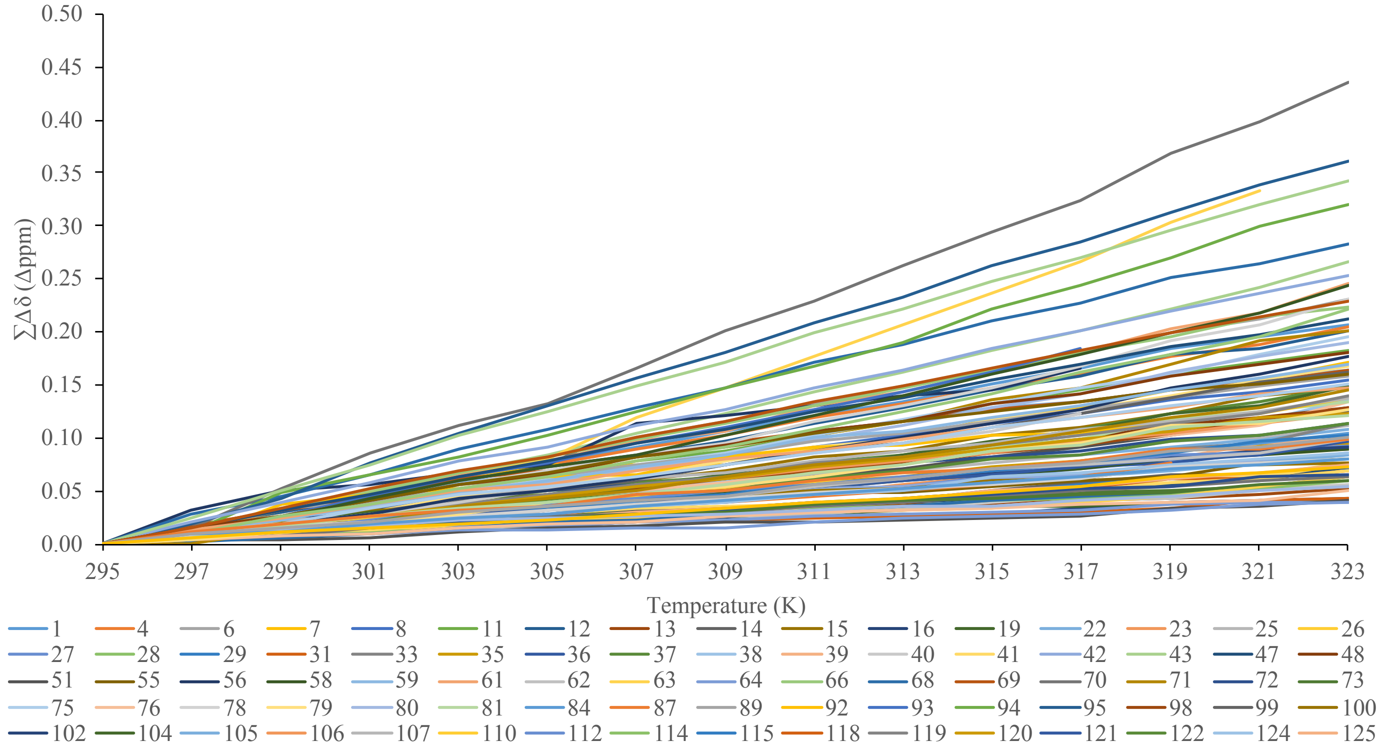

Figure S.2. Temperature dependence of ∑Δδ for all assigned residues. Trends for residues are coloured as shown in the legend. The starting temperature is 295 K and therefore has no cumulative Δδ distance.

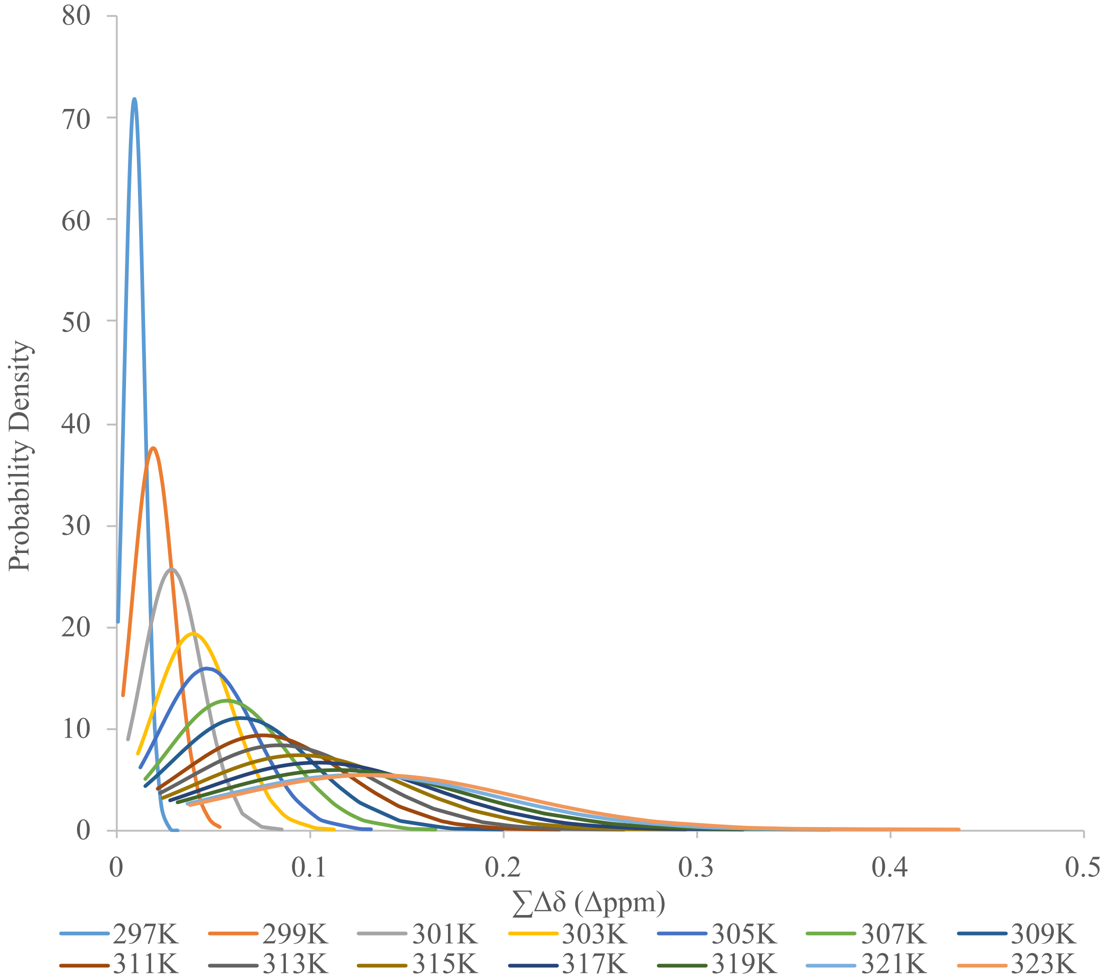

Figure S.3. Normal Distribution of ∑Δδ at Each Temperature Point. The temperature for each distribution is indicated in the legend.

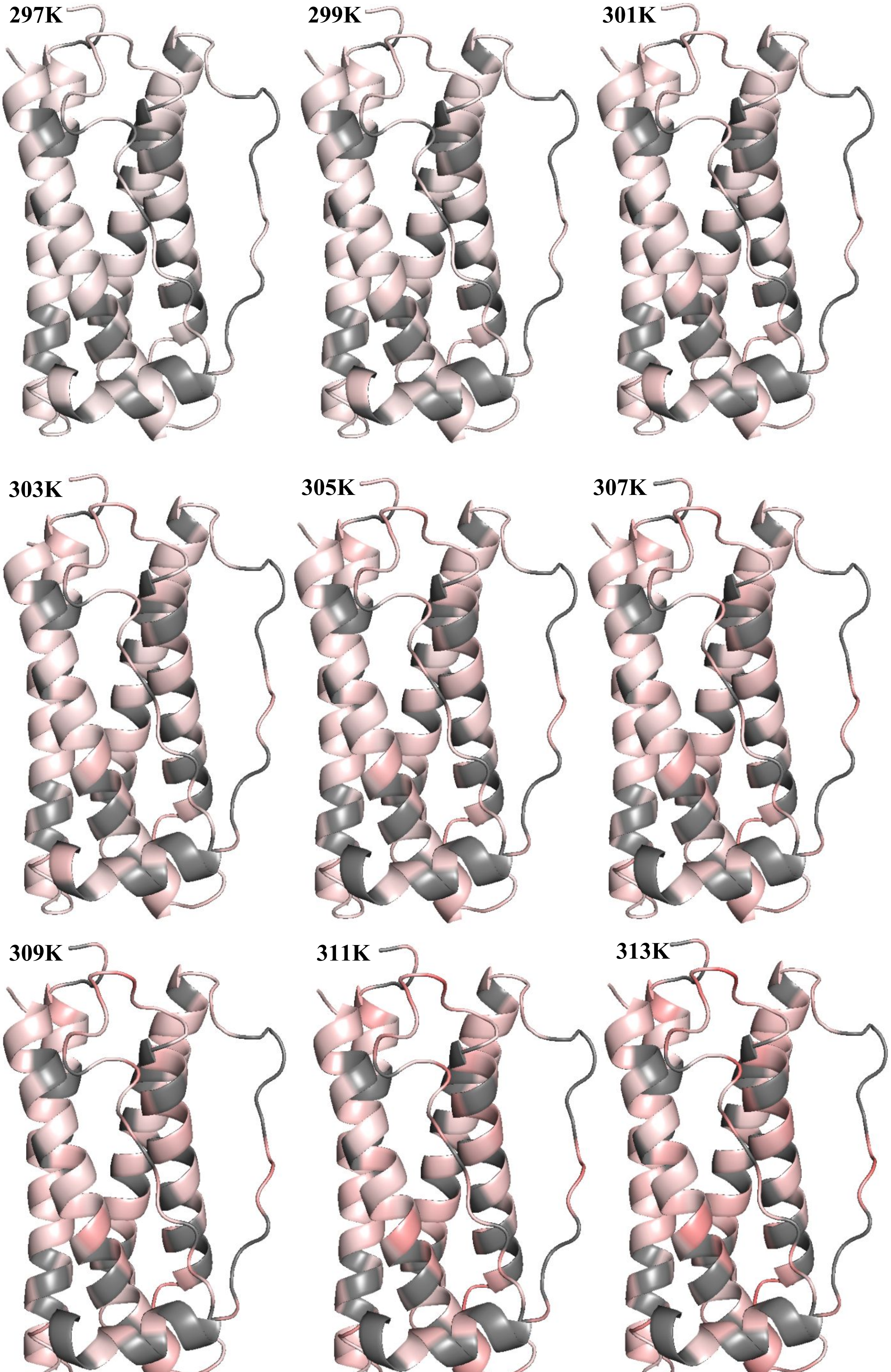
297K
299K
301K
303K
305K
307K
309K
311K
313K

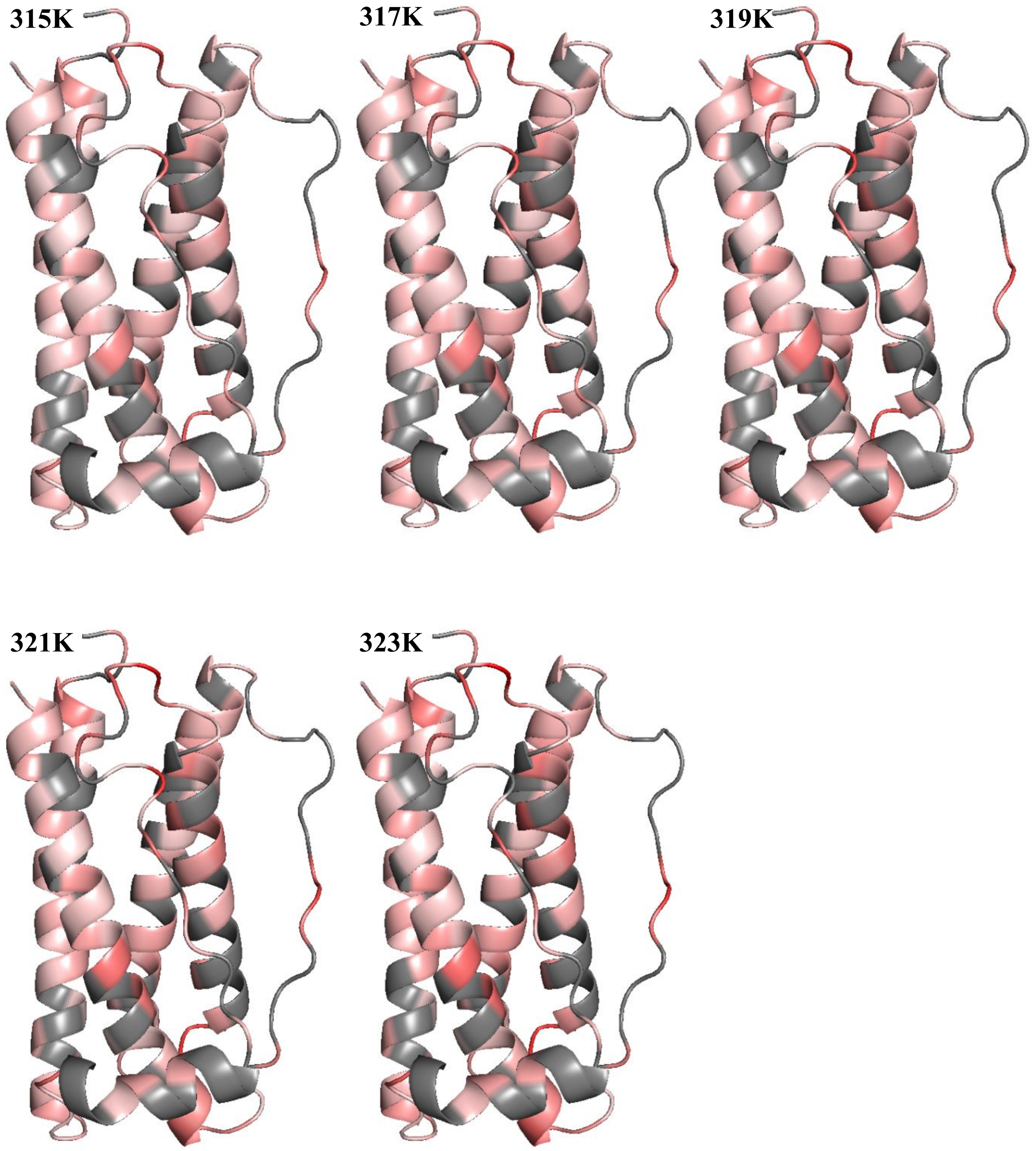

Figure S.4. Mapping ∑Δδ on G-CSF Structure

G-CSF (PDB:2D9Q) coloured according to ∑Δδ at their respective temperature. Red signifies the highest ∑Δδ value (0.435 Δppm), and white signifies the lowest ∑Δδ value (0.000425 Δppm) observed for the whole data set across the thermal melt, while grey represents unassigned residues. Unassigned resides are coloured grey. Residues T1 to A6 are missing in this structure.

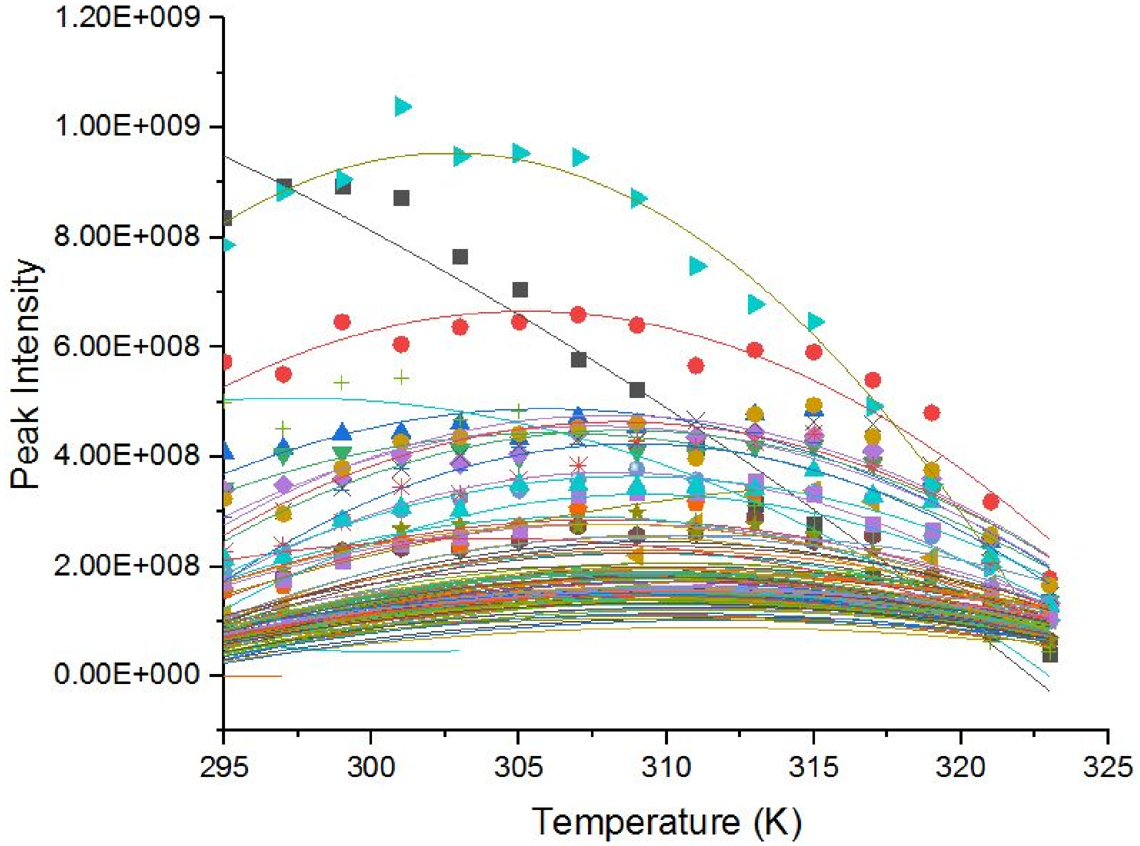

Figure S.5. Raw PI Data with Temperature.

Each different coloured line represents a different assigned residue.

Linearity of peak trajectories Δδ are given in Table S.1, where 68 trajectories are considered as linear when they have an R^2 value over 0.9 (based on their 1-H and 15-N δ values). The remaining 44 residues for which linearity was calculated are non-linear. Accompanying this table is the structure of G-CSF, highlighting non-linear (red scale), linear (white) and unassigned (grey) residues (Figure S.6A).

Table S.1. Linear trajectories are defined as those with an R^2 >0.9 and are coloured white. Non-linear trajectories are coloured red.

Residue Number	Linearity	R-Squared	Residue Number	Linearity	R-Squared	Residue Number	Linearity	R-Squared
1	Linear	0.993	71	Linear	0.992	145	Linear	0.994
4	Linear	0.993	72	Linear	0.923	146	Linear	0.969
6	Linear	0.992	73	Non-Linear	0.387	149	Non-Linear	0.052
8	Linear	0.996	75	Linear	0.972	150	Non-Linear	0.324
11	Linear	0.935	76	Non-Linear	0.886	151	Non-Linear	0.575
12	Non-Linear	0.780	78	Linear	0.979	153	Linear	0.927
13	Non-Linear	0.868	79	Linear	0.987	155	Non-Linear	0.790
14	Non-Linear	0.439	80	Linear	0.916	156	Linear	0.992
15	Linear	0.996	81	Linear	0.990	157	Non-Linear	0.862
16	Non-Linear	0.702	84	Linear	0.959	158	Non-Linear	0.856
19	Non-Linear	0.520	87	Linear	0.929	159	Linear	0.956
22	Linear	0.978	89	Linear	0.971	160	Non-Linear	0.869
23	Non-Linear	0.576	92	Linear	0.938	161	Non-Linear	0.530
25	Linear	0.922	93	Linear	0.990	163	Non-Linear	0.298
26	Non-Linear	0.793	94	Linear	0.944	164	Linear	0.944
27	Non-Linear	0.678	95	Linear	1.000	165	Linear	0.962
28	Linear	0.912	98	Non-Linear	0.872	166	Non-Linear	0.135
29	Linear	0.977	99	Linear	0.961	169	Linear	0.965
31	Non-Linear	0.071	100	Non-Linear	0.581	170	Non-Linear	0.121
33	Linear	0.935	102	Linear	0.961	171	Non-Linear	0.556
35	Linear	0.940	104	Linear	0.954	172	Non-Linear	0.586
36	Non-Linear	0.818	105	Linear	0.916	173	Non-Linear	0.897
37	Linear	0.967	106	Linear	0.989			
38	Non-Linear	0.838	107	Linear	0.975			
39	Non-Linear	0.408	110	Linear	0.983			
40	Non-Linear	0.679	112	Non-Linear	0.832			
41	Non-Linear	0.848	114	Non-Linear	0.724			
42	Linear	0.928	115	Linear	0.983			
43	Linear	0.914	118	Linear	0.960			
47	Non-Linear	0.758	119	Linear	0.983			
48	Linear	0.985	120	Linear	0.982			
51	Linear	0.970	121	Linear	0.921			
55	Non-Linear	0.024	122	Linear	0.978			
56	Non-Linear	0.188	124	Linear	0.914			
58	Non-Linear	0.845	125	Linear	0.977			

59	Linear	0.923	126	Linear	0.987				
61	Linear	0.990	127	Linear	0.949				
62	Non-Linear	0.378	133	Linear	0.997				
63	Linear	0.980	134	Linear	0.998				
64	Non-Linear	0.686	135	Linear	0.997				
66	Linear	0.998	139	Linear	0.968				
67	Non-Linear	0.736	141	Non-Linear	0.168				
68	Linear	0.993	142	Linear	0.993				
69	Non-Linear	0.517	143	Linear	0.954				
70	Linear	0.985	144	Non-Linear	0.636				

A. **B.**

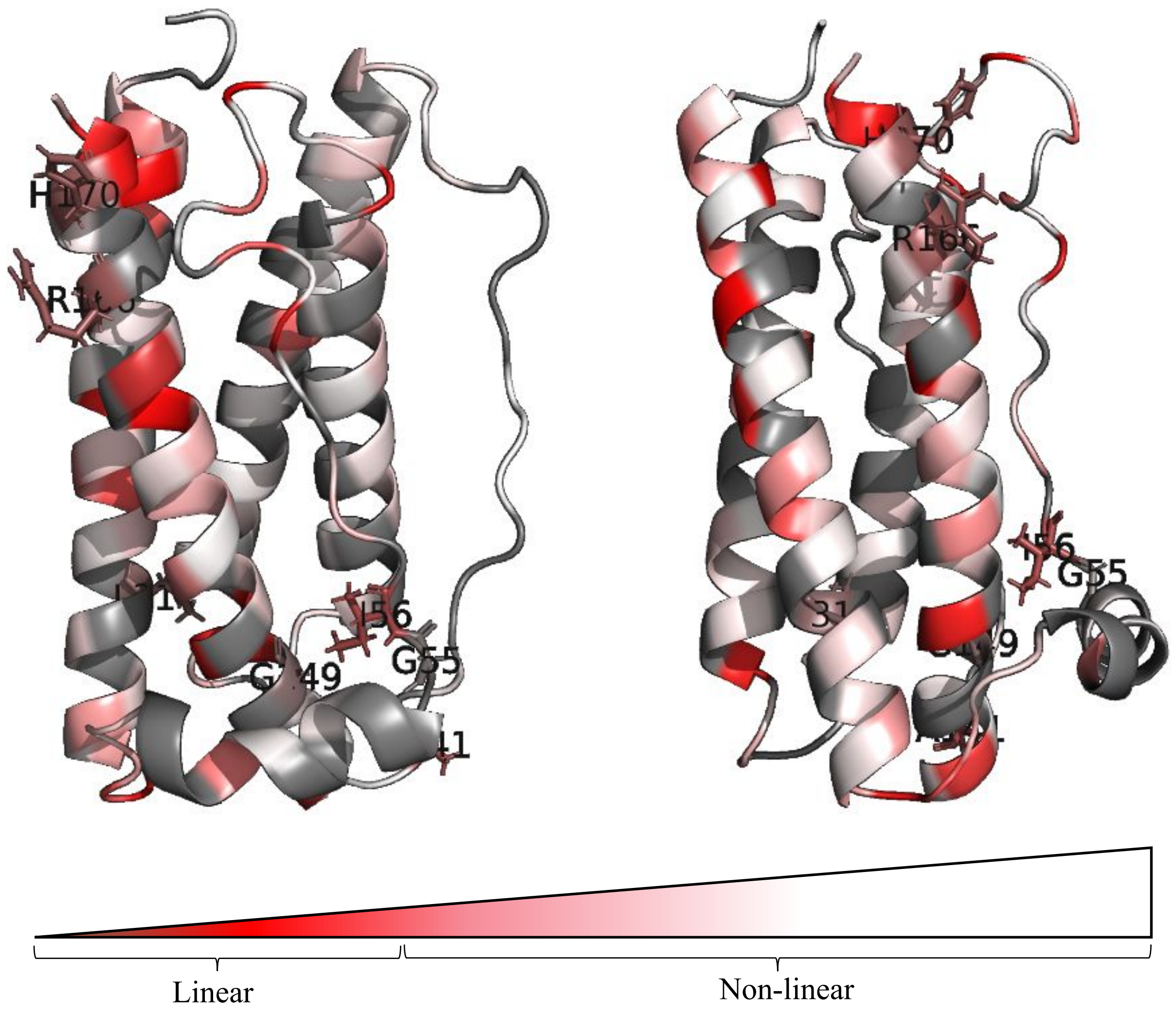

Figure S.6. Determining Peak Trajectory Linearity. Trajectory linearity for residues is coloured on a white to red scale, with darker red representing a lower R^2 value and so more non-linearity, white representing higher linearity. **A)** front (left-hand-side) and **B)** side (right-hand-side) view of PDB:2D9Q. Extremely non-linear residues (R^2 <0.3) are labelled and shown as sticks. Unassigned residues are light grey.

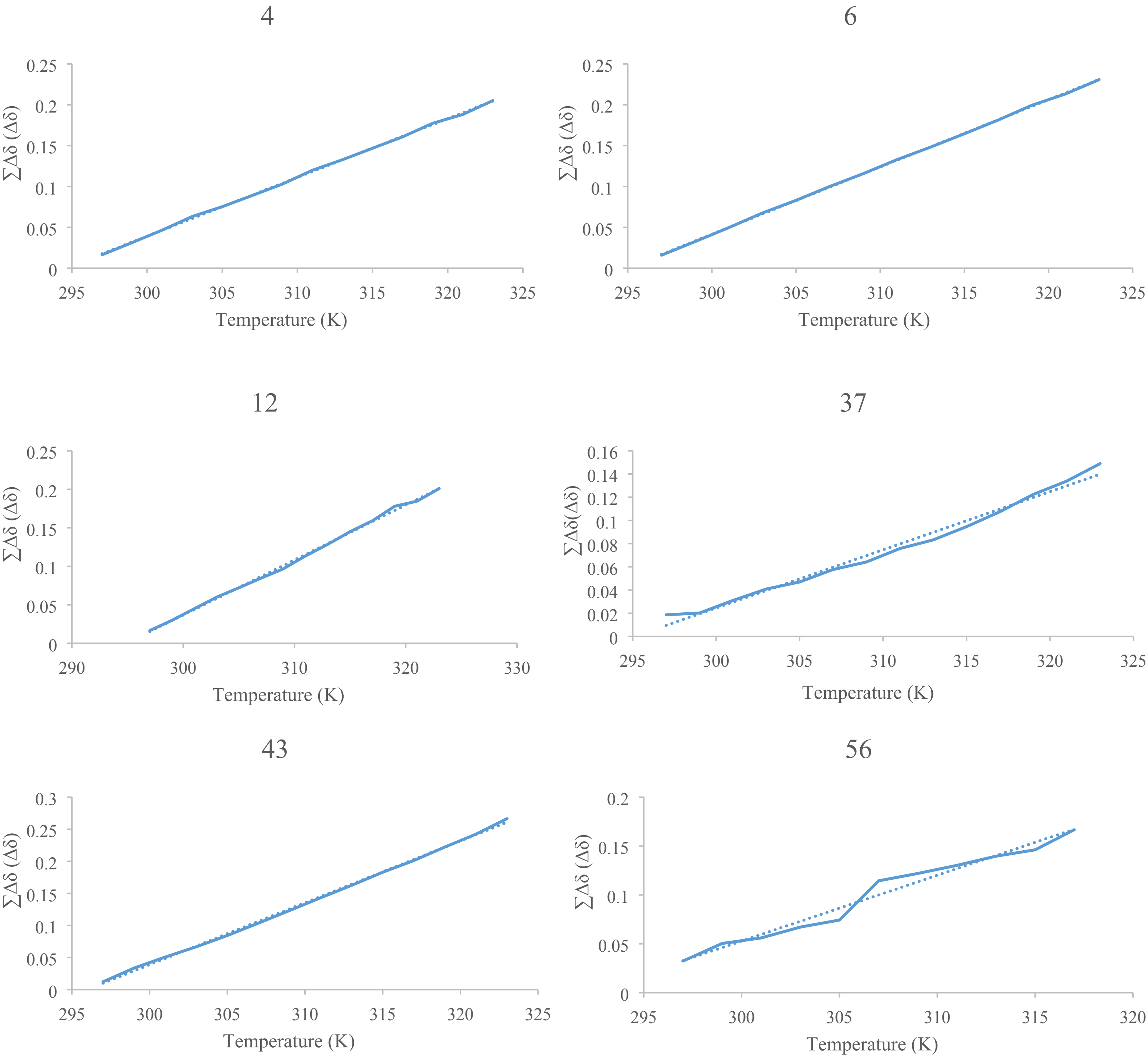
4
6
12
37
43
56
∑Δδ (Δδ)
∑Δδ(Δδ)
Temperature (K)
0
0.02
0.04
0.05
0.06
0.08
0.1
0.12
0.14
0.15
0.16
0.2
0.25
0.3
290
295
300
305
310
315
320
325
330

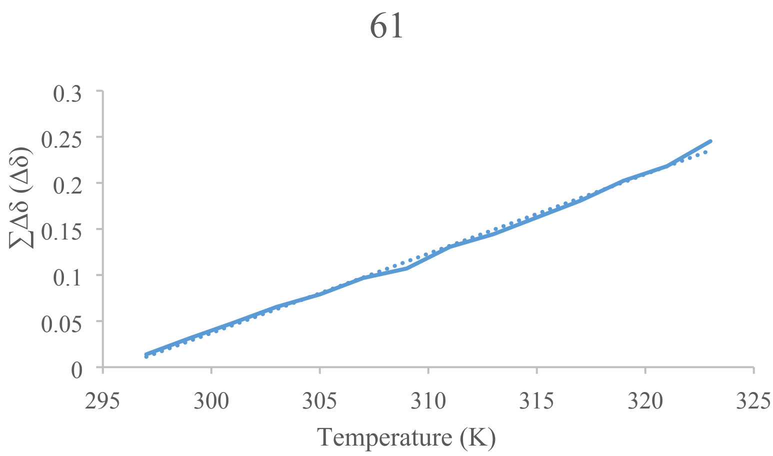
61
∑Δδ (Δδ)
0.3
0.25
0.2
0.15
0.1
0.05
0
295
300
305
310
315
320
325
Temperature (K)

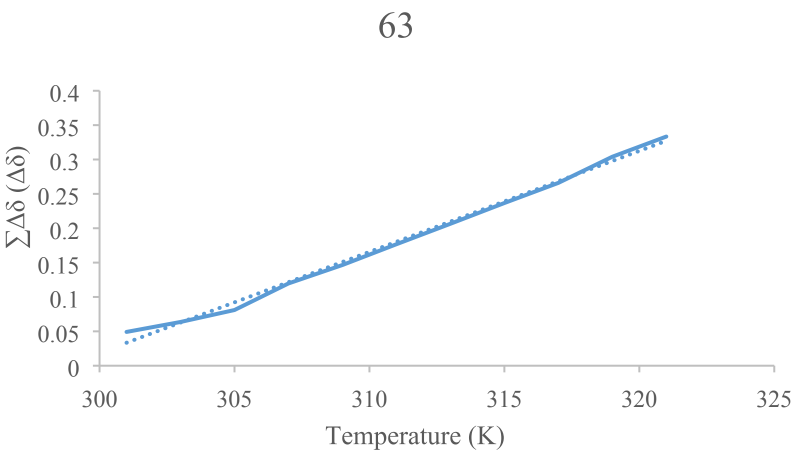
63
∑Δδ (Δδ)
0.4
0.35
0.3
0.25
0.2
0.15
0.1
0.05
0
300
305
310
315
320
325
Temperature (K)

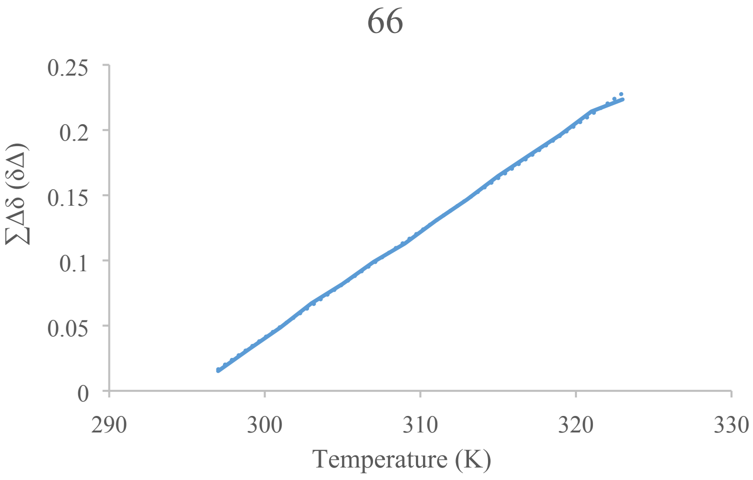
66
∑Δδ (δΔ)
0.25
0.2
0.15
0.1
0.05
0
290
300
310
320
330
Temperature (K)

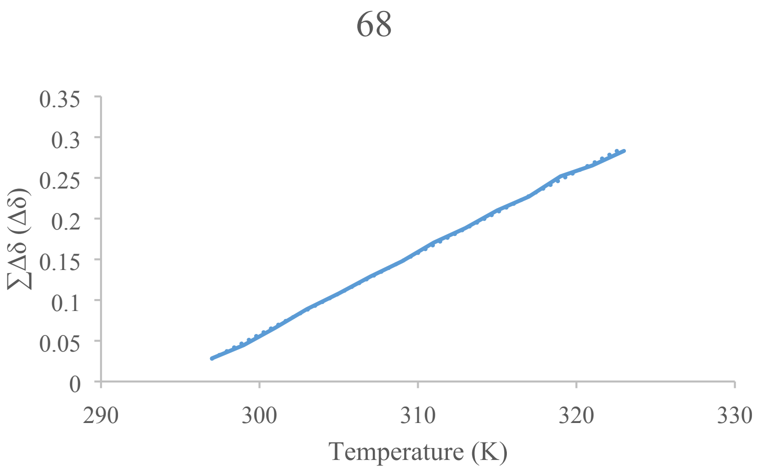
68
∑Δδ (Δδ)
0.35
0.3
0.25
0.2
0.15
0.1
0.05
0
290
300
310
320
330
Temperature (K)

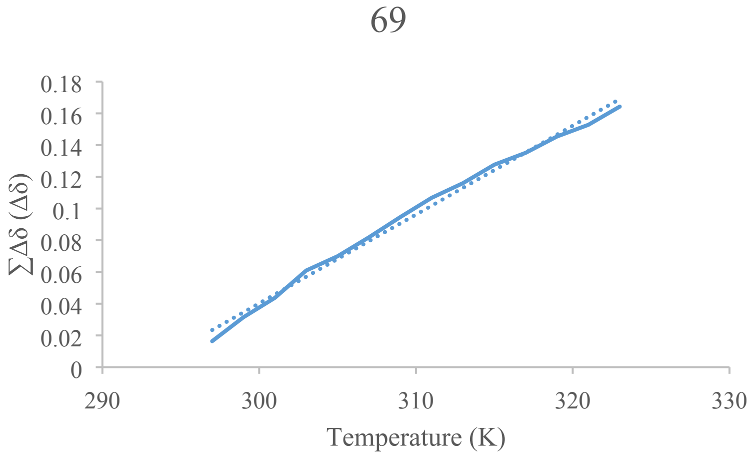
69
∑Δδ (Δδ)
0.18
0.16
0.14
0.12
0.1
0.08
0.06
0.04
0.02
0
290
300
310
320
330
Temperature (K)

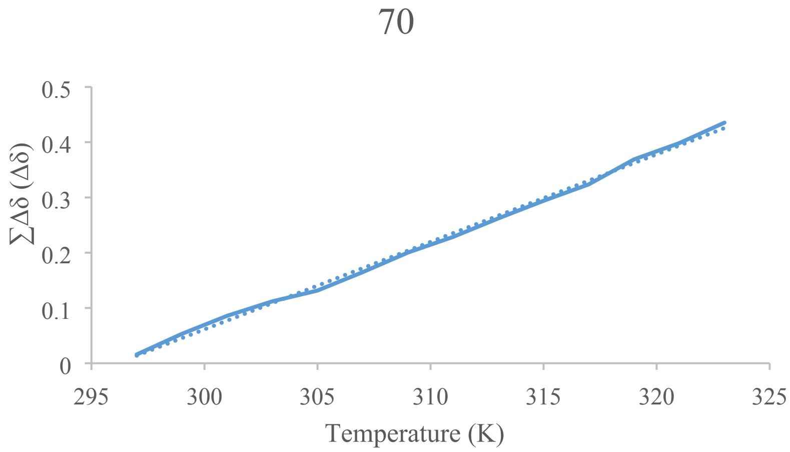
70
∑Δδ (Δδ)
0.5
0.4
0.3
0.2
0.1
0
295
300
305
310
315
320
325
Temperature (K)

78

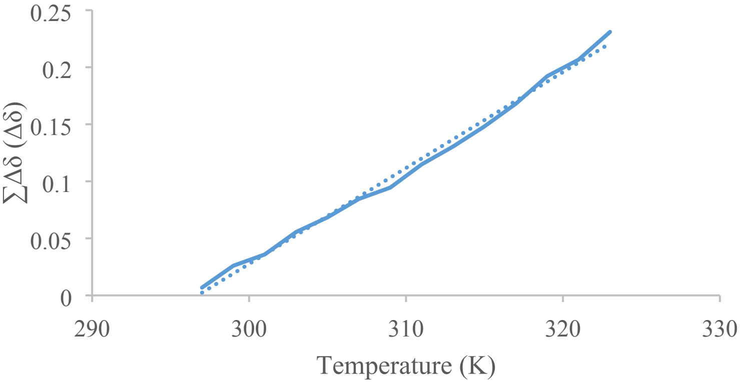

89

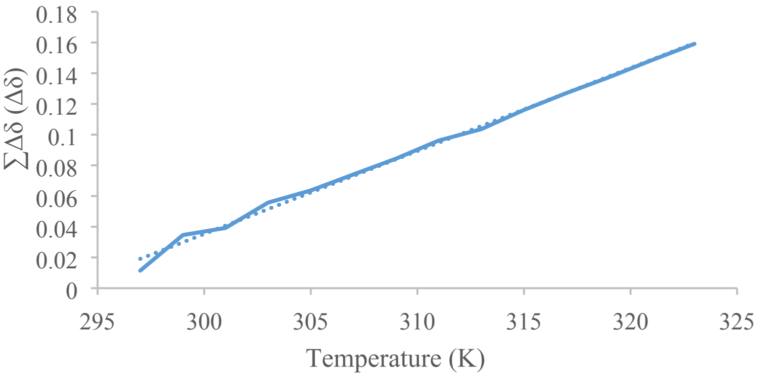

92

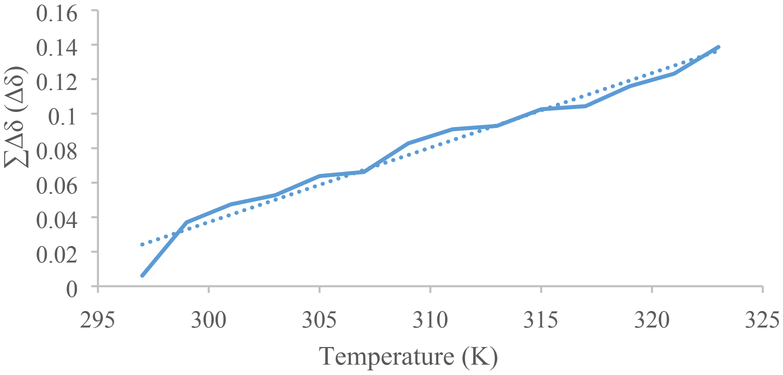

93

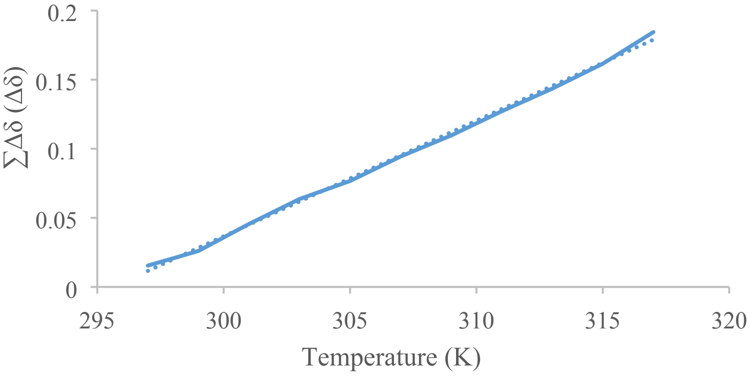

94

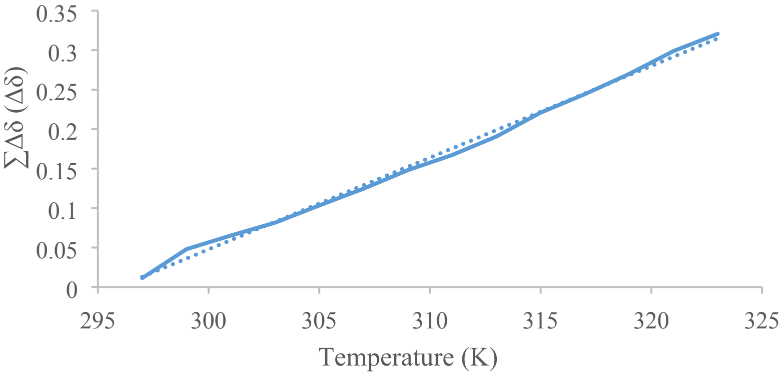

95

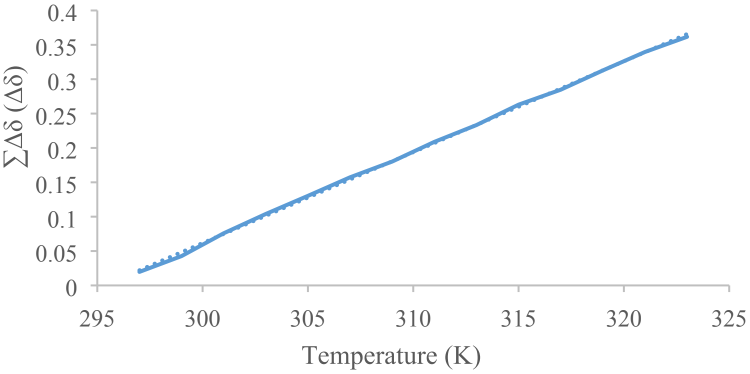

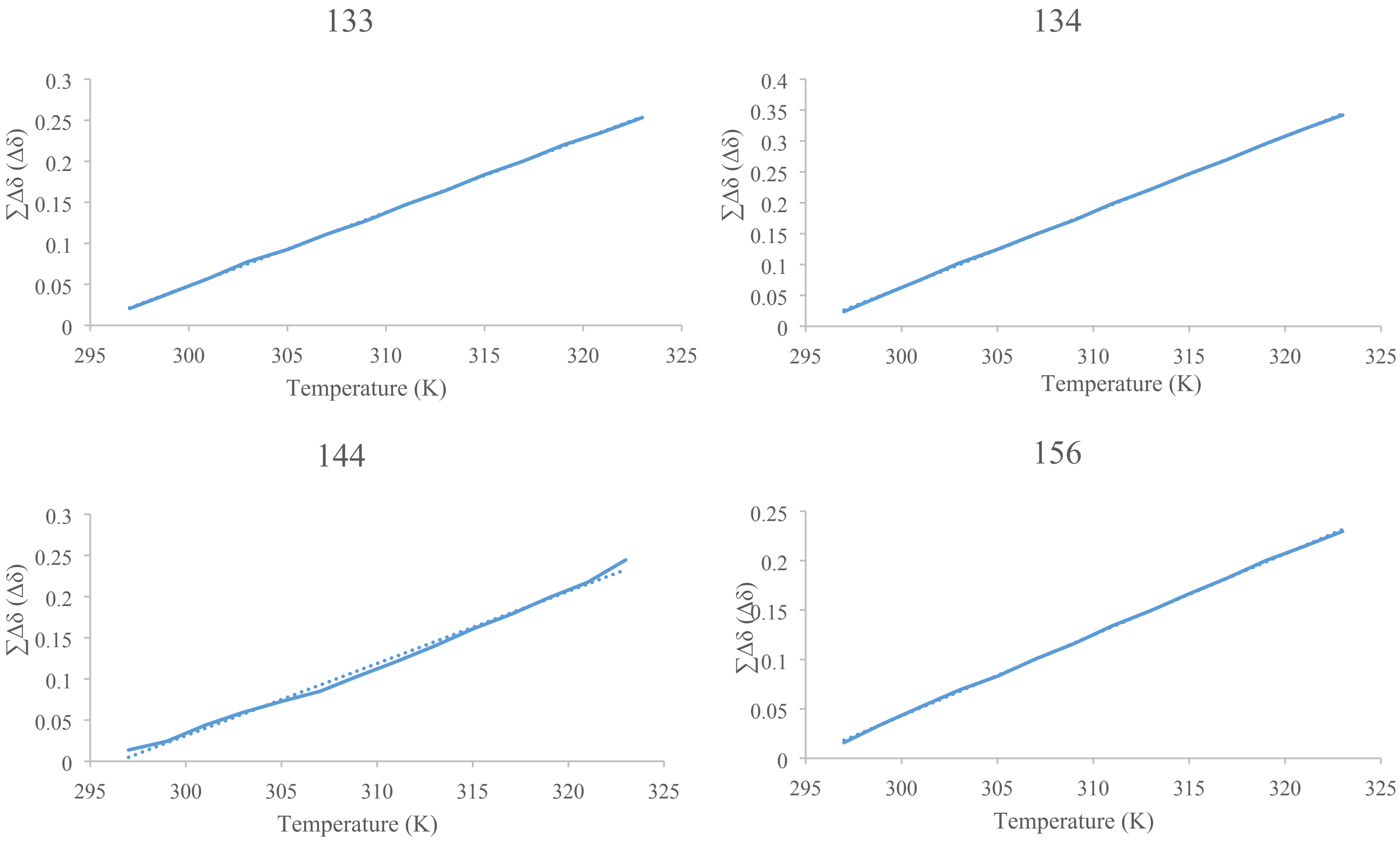

Figure S.7. Monitoring ∑Δδ for 90th Percentile Residues. Individual ∑Δδ vs temperature plots for residues in the top 90th percentile of ∑Δδ are shown. Each ∑Δδ vs temperature plot is accompanied by the respective y= mx + c equation.

Although all ∑Δδ vs temperature plots are linear, deviations from the y = mx + c line differs for all residues. Large deviations from this line may indicate significant transitions at certain

temperatures. Therefore, to probe these residues that possibly experience significant transitions, R^2 values are calculated for all residues. Those with a value in the lower quartile (under 0.99) are considered to have a non-linear "$\sum\Delta\delta$-temperature relationship" and are tabulated in Table S.2A. The temperature point at which these residues (red) and those in the $\sum\Delta\delta$ 90th percentile (green) experience their largest deviation from their y = mx + c line is shown in Table S.2B. Both of these residue categories are combined to examine how residues experiencing significant environmental changes may influence conformational transitions for other residues. Residues I56 and A37 are in the $\sum\Delta\delta$ 90th percentile and have R^2 values in the lower quartile. A "temperature line" illustrating residues in Table S.2B on the structure of G-CSF is given in Figure S.8A, showing that when $\sum\Delta\delta$ 90th percentile residues (in green) experience their largest deviation, as do proximal residues in the lower quartile for R^2 (in red).

Table S.2A. R^2 values for linearity of $\sum\Delta\delta$ vs temperature for each residue.

**Residue**	**R^2**
19	0.930
150	0.950
100	0.961
92	0.967
56	0.972
141	0.973
151	0.975
87	0.978
47	0.980
41	0.981
55	0.981
16	0.984
33	0.985
59	0.985
31	0.985
26	0.986
163	0.986
37	0.986
64	0.986
166	0.987
127	0.987
73	0.987
104	0.989
14	0.989
149	0.989

Table S.2B. Temperatures at which residues become non-linear in their trajectories for $\sum\Delta\delta$ vs temperature for all residues with R^2 <0.99. Those in the $\sum\Delta\delta$ 90th percentile for lowest R^2

are highlighted in green. Non-linear residues in the lower quartile for R^2 are highlighted in red to match the shading in Figure S8.

	Temperature (K)													
	**297**	**299**	**301**	**303**	**305**	**307**	**309**	**311**	**313**	**315**	**317**	**319**	**321**	**323**
**Residues**	69	94		134	63	56	92		33	95	31	6	4	43
	126	89		156	55	144	93			16		12	66	61
	19			149		59	127			41		14	73	68
	64						151			47		87	163	70
	100									141				133
										166				78
														37
														26
														104
														150

A.

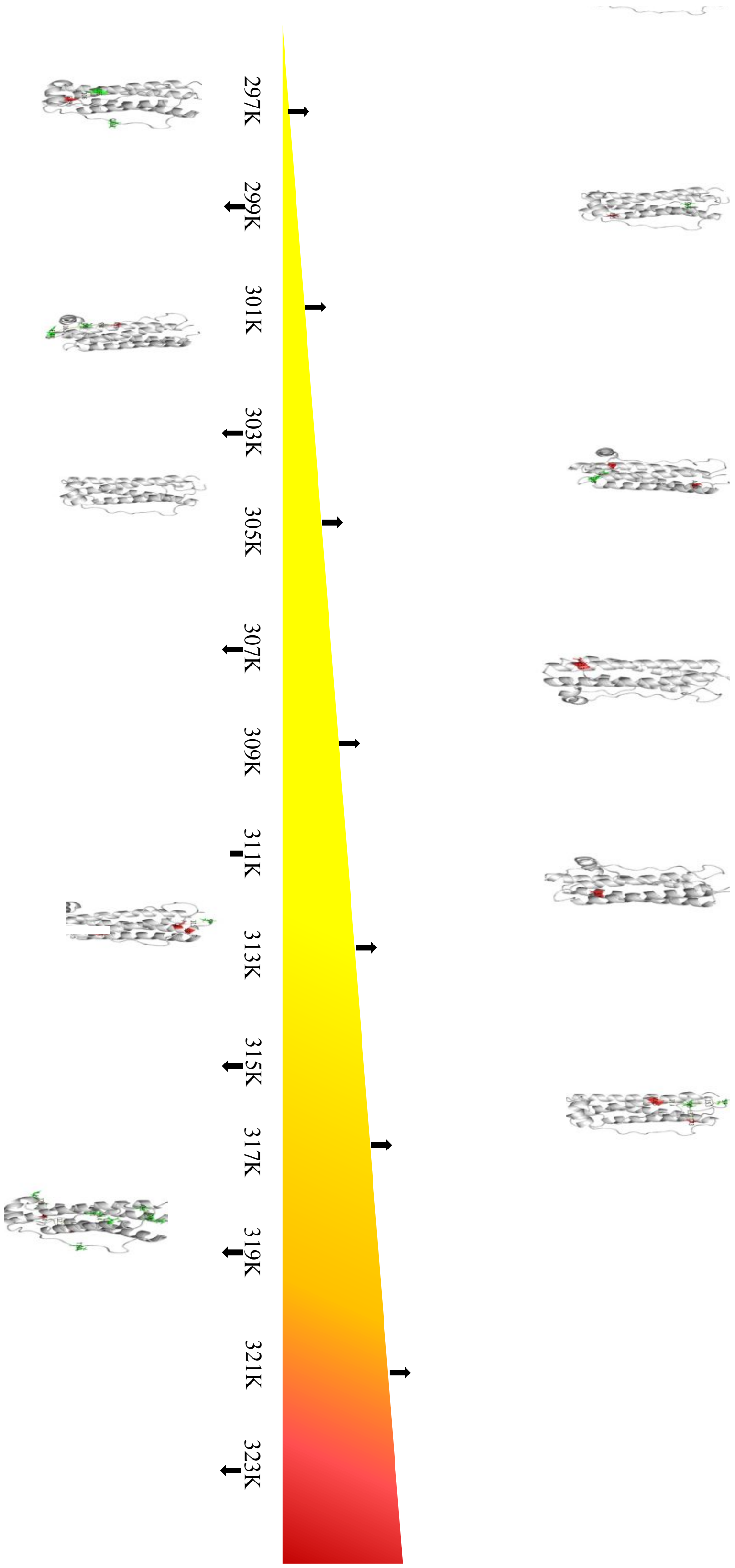

Figure S.8. Schematic temperature line. Residues are highlighted at the temperature at which non-linearity occurred for $\sum\Delta\delta$ 90th percentile residues (in green) and residues in the lower quartile for R^2 (in red). Residue S7 was used in place of G4 and A6.

Percentage Change in PI

Percentage change was calculated using the PI value at the start of the thermal melt and at the maximum point, which is typically around 309 K.

Table S.3. Percentage increases in PI (in white columns) are given for all assigned residues (in grey columns), which is the only residue to show a decrease in PI at the start of the melt. Top 15 percentage increases are highlighted in yellow.

Percentage Increase in ΔPI							
**Residue Number**	**%Increase**	**Residue Number**	**%Increase**	**Residue Number**	**%Increase**	**Residue Number**	**%Increase**
51	297	115	124	16	95	135	54
43	241	14	123	76	95	156	53
153	229	124	122	134	95	72	51
70	227	99	121	15	94	64	50
94	210	144	121	25	91	163	50
55	203	62	121	119	91	81	47
36	202	107	120	69	90	27	43
59	202	35	119	29	89	133	41
38	192	155	119	157	89	169	36
56	190	31	112	170	89	8	36
126	184	47	112	164	88	48	32
89	183	39	110	61	86	7	23
33	181	66	110	73	85	6	19
41	166	171	110	42	84	50	18
106	157	142	109	58	84	4	15
71	156	114	109	166	83	120	9
93	151	100	108	102	83	1	7
78	150	95	107	172	80		
63	149	87	106	68	78		
37	144	84	106	26	78		
28	143	80	105	141	77		
145	137	150	104	19	77		
92	137	127	102	12	75		
161	134	159	100	173	75		
151	134	105	100	160	72		
40	134	79	100	112	71		
146	134	110	99	75	70		
158	133	118	98	22	70		
165	133	149	98	104	56		
98	132	121	97	139	56		
13	130	125	96	143	55		
11	129	122	96	23	55		

A.

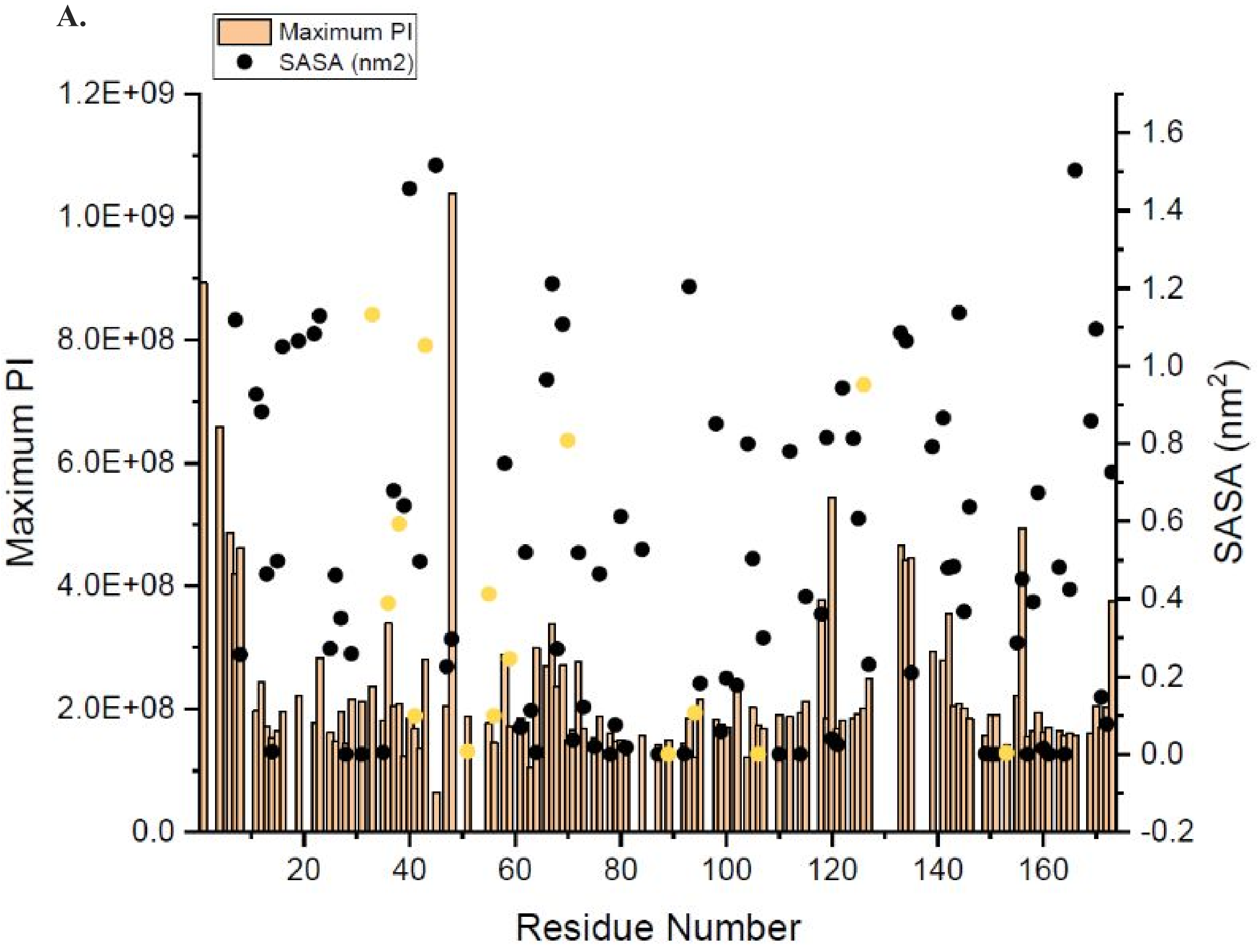

Figure S.9. Top 15 Residues with Highest Percentage Increase in ΔPI. Maximum PI vs. SASA. Residues in the sub-clusters have their SASA highlighted yellow.

Mapping Significant Structural Changes on to G-CSF

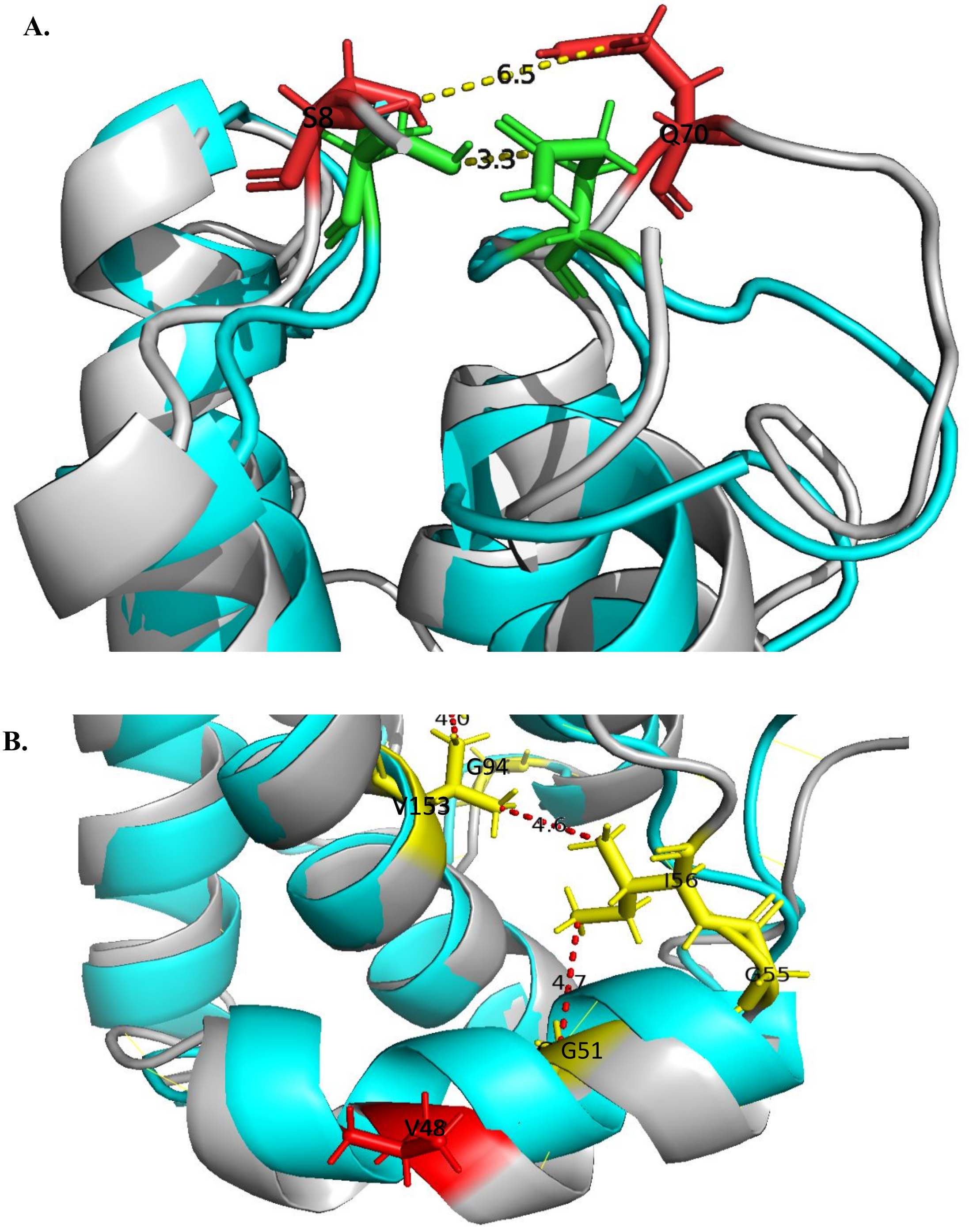

Figure S.10. Proximity of residue S8 to Q70 and exposure of Sub-cluster 2 by V48.

Relaxed (cyan) and unrelaxed (grey) G-CSF structures are overlaid. **A.** S8 and Q70 are highlighted green in the relaxed structure and red in the unrelaxed structure. Distance between both of these residues is 3.3 Ǻ in the relaxed structure and 6.5 Ǻ in the unrelaxed structure. **B.** shows residues in sub-cluster 2 (yellow). Distance between these residues are indicated along red dotted lines. Residue V48 is highlighted red.

APRs

AmylPred 2 (Tsolis *et al*., 2013) employs a consensus method to identify aggregation prone regions (APRs) APRs, combing results from several different software mentioned in Figure S.12. The 5 different APR consensus regions predicted by AmylPred 2 based on successful hits from at least 5 out of the 10 software are V48-S53, G81-L89, T115-Q119, L152-L157 and S159-L171. These regions are respectively coloured as green, red, blue and yellow (for L152-L157 and S159-L171 since they are so close in sequence) in. Helix D has the largest hotspot of APRs (coloured yellow).

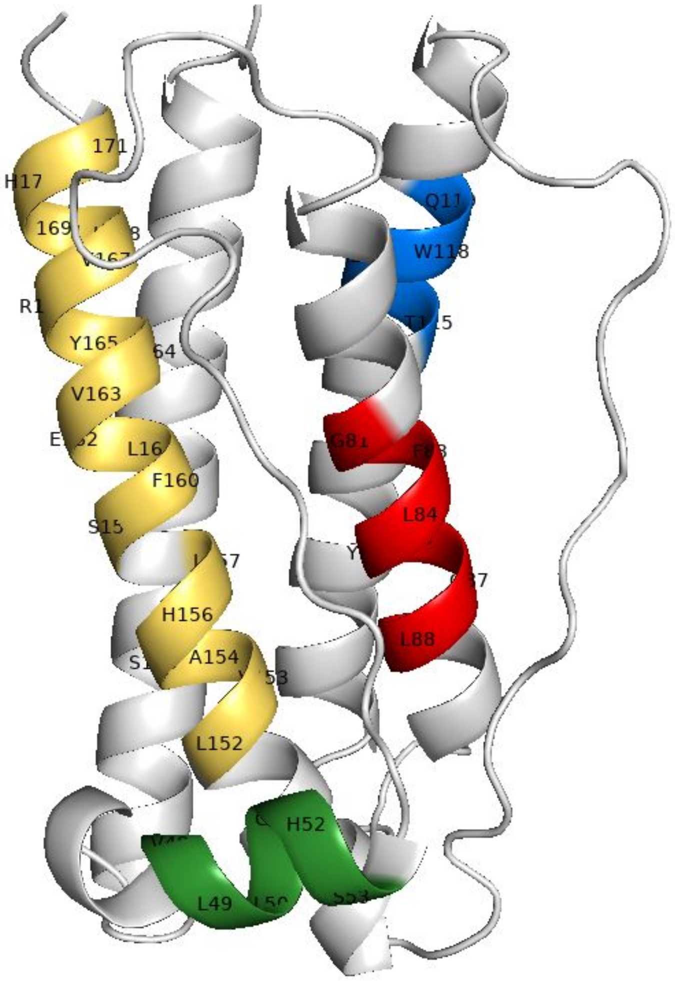

Consensus Software
AGGRESCAN
Amyloidogenic Pattern
Average Packing Density
Beta-strand contiguity
Hexapeptide Conf. Energy
NetCSSP
Pafig
SecStr
TANGO
WALTZ

Figure S.11. Identifying APRs. Five different Consensus APRs determined from AmylPred 2 are coloured yellow, red, green and blue. Two regions are coloured yellow because they are only one residue apart. The ten APR scanning software used for the consensus are shown in the accompanying table.

25mM Arginine

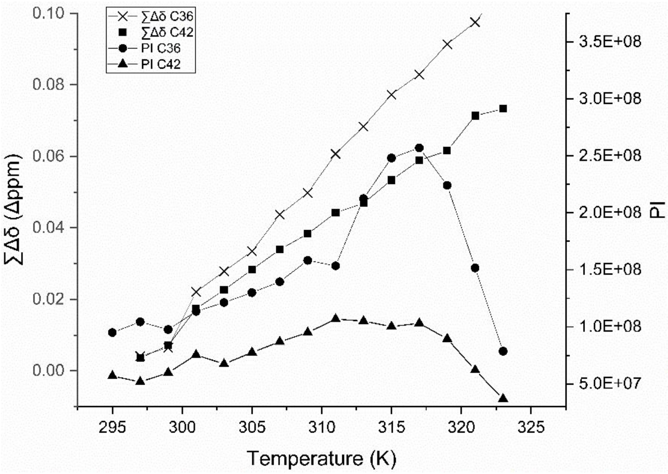

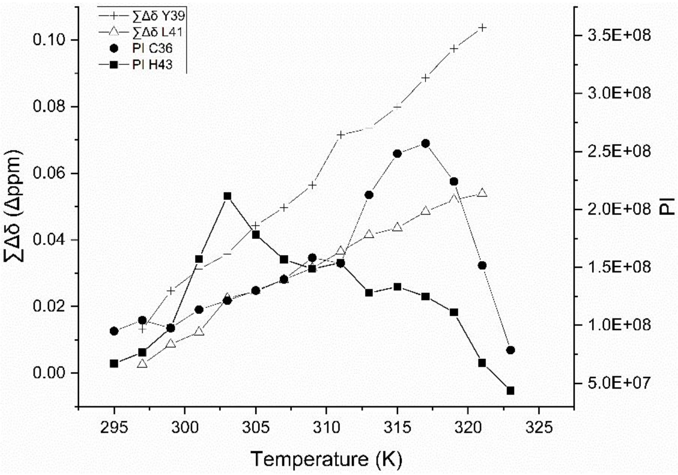

25mM Histidine

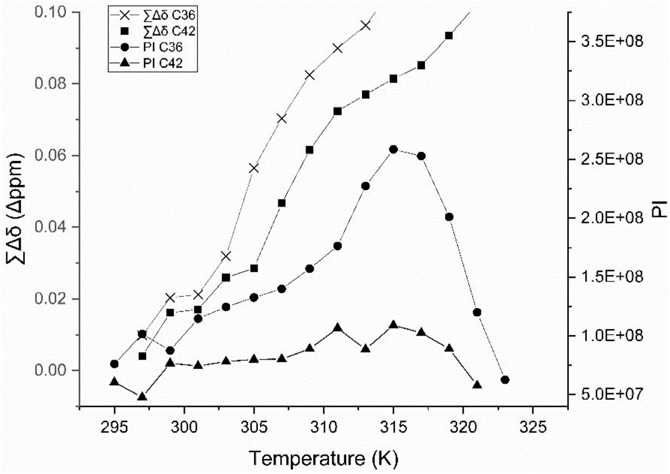

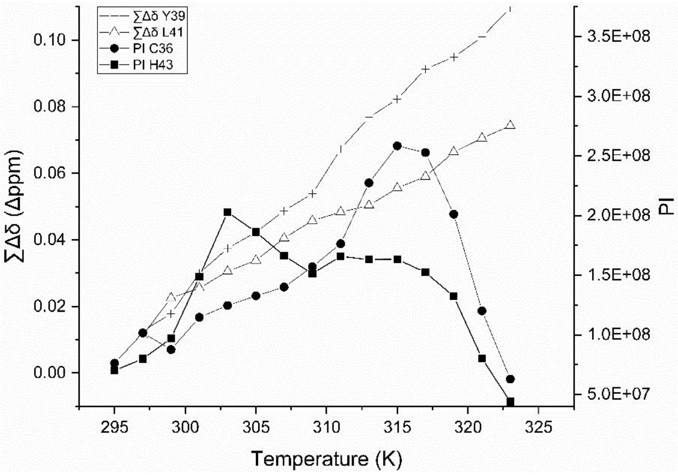

12.5mM Phenylalanine

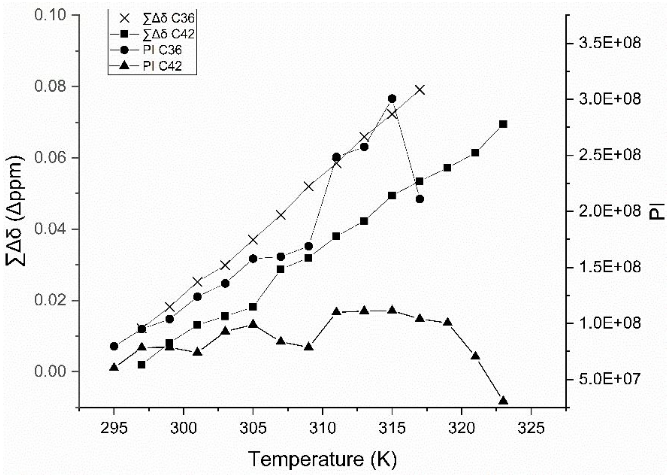

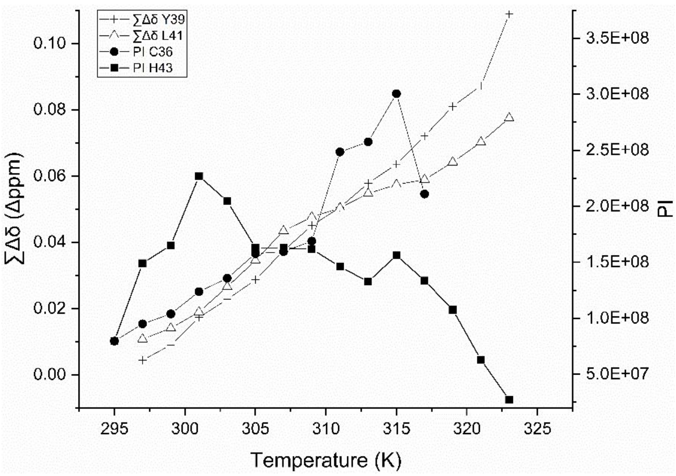

Figure S.12. Comparing the "Switch Mechanism" in Different Buffers

NMR observables compared for same residues highlighted in Figure 7B (left-hand-side) and C (right-hand-side), for 0.09 mM WT G-CSF (pH 4.25, 50 mM sodium acetate) with the addition of 25 mM Arginine, 25 mM Histidine and 12.5 mM Phenylalanine.
